# Neutrophil‐Mimicking Nanomedicine Eliminates Tumor Intracellular Bacteria and Enhances Chemotherapy on Liver Metastasis of Colorectal Cancer

**DOI:** 10.1002/advs.202504188

**Published:** 2025-05-28

**Authors:** Yanan Niu, Xu Zhao, Yong Li, Xiaoya Ma, Weifeng Yang, Jie Ma, Wanglin Li, Wei Yuan

**Affiliations:** ^1^ State Key Laboratory of Molecular Oncology National Cancer Center/Cancer Hospital Chinese Academy of Medical Sciences Peking Union Medical College Beijing 100021 P. R. China; ^2^ Department of Thoracic Surgery National Cancer Center/Cancer Hospital Chinese Academy of Medical Sciences Peking Union Medical College Beijing 100021 P. R. China; ^3^ Department of Gastrointestinal Surgery Huadu District Peoples' Hospital of Guangzhou 48 Xinhua Road, Huadu District Guangzhou 510800 P. R. China; ^4^ Department of Biotherapy Beijing Hospital National Center of Gerontology Institute of Geriatric Medicine Chinese Academy of Medical Sciences Graduate School of Peking Union Medical College Beijing 100730 P. R. China

**Keywords:** colorectal cancer, liver metastasis, nanomedicine, tumor microbiome

## Abstract

*Fusobacterium nucleatum* (*Fn*) enrichment has been identified in colorectal cancer and its liver metastases. In this study, we found that *Fn* predominantly accumulated within colorectal cancer cells, correlating with colorectal cancer liver metastasis. Clinically, the administration of high doses of antibiotics and chemotherapeutic agents can disrupt the balance of the host microbiota. To address this clinical challenge, metronidazole (MTI) and oxaliplatin (OXA) are encapsulated within poly (lactic‐co‐glycolic acid) (PLGA) nanoparticles. Neutrophil membrane vesicles are extracted from murine bone marrow and coated with these nanoparticles (NM@PLGA‐MTI‐OXA), creating neutrophil‐mimetic nanoparticles with dual targeting capabilities for antibacterial and anticancer purposes. The neutrophil membrane coating, compared with free drugs, is found to enhance nanoparticle uptake by tumor cells, facilitating intracellular bacterial elimination and tumor cell death. Further experiments reveal that NM@PLGA‐MTI‐OXA reverses the *Fn*‐induced epithelial‐mesenchymal transition (EMT) in tumor cells during metastasis and remodels the immunosuppressive microenvironment, suppressing colorectal cancer and liver metastasis development while minimizing broad‐spectrum damage to the commensal microbiota.

## Graphical abstract

*Fusobacterium nucleatum (Fn)* enrichment has been identified in colorectal cancer and its liver metastases. This study develops neutrophil‐mimicking nanoparticles with multiple targeting capabilities encapsulating metronidazole and oxaliplatin for antibacterial and anticancer purposes. This approach reverses the epithelial mesenchymal transition process caused by *Fn* and reshapes the immunosuppressive microenvironment to suppress the development of liver metastasis, meanwhile avoiding normal microbial communities’ damage.

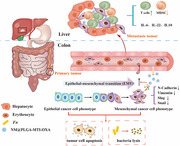

## 1 Introduction

Colorectal cancer (CRC) is the third most common malignancy worldwide and the second leading cause of cancer‐related mortality.^[^ ^1^, ^2^ ^]^ The majority (80%–90%) of liver metastases from CRC are initially considered unresectable, significantly contributing to patient mortality.^[^ ^3^, ^4^ ^]^ Chemotherapy remains the mainstay of treatment for metastatic colorectal cancer.^[^ ^5^ ^]^ However, the heterogeneity and molecular diversity of CRC often lead to chemotherapy resistance, which is limited by systemic toxicity, low patient selectivity, and relatively low response rates.^[^ ^6^, ^7^ ^]^

In recent years, advances in detection techniques and a deeper understanding of the tumor microenvironment have yielded increasing evidence confirming the presence of intratumoral bacteria.^[^ ^8^, ^9^, ^10^, ^11^ ^]^ *Fusobacterium nucleatum* (*Fn*), a common member of the human oralmicrobiome, canspecifically bind to Gal‐GalNac, which is highly expressed on colorectal cancer cells, via its surface protein FAP2. This binding allows *Fn* to colonize CRC tissue sites^[^ ^12^, ^13^ ^]^ and induce chemoresistance to agents such as oxaliplatin (OXA) and 5‐fluorouracil (5‐FU).^[^ ^14^ ^]^ Furthermore, *Fn* has been detected and cultured from human liver metastatic tissues, with its genotype identical to that of matched primary colorectal cancer tissues, suggesting its potential involvement in distant metastasis of CRC cells.^[^ ^15^ ^]^ Several studies have suggested that *Fn* could promote CRC metastasis through various pathways.^[^ ^16^ ^]^ For instance, *Fn* can stimulate tumor cells to produce exosomes rich in miRNA, which are transported to uninfected cells to promote metastasis.^[^ ^17^ ^]^ Besides, it has been reported that *Fn* could enhance the metastatic ability of CRC cells by reducing METTL3‐mediated m^6^A modification.^[^ ^18^ ^]^ Importantly, *Fn* not only promotes CRC progression but also plays a crucial role in distant CRC metastasis, making it a potential key target for the treatment of colon cancer and its metastasis.

Metronidazole (MTI), a commonly used antibiotic against anaerobic bacteria, can effectively reduce the abundance of *Fn*.^[^ ^15^, ^19^ ^]^ Unfortunately, gut dysbiosis induced by MTI can lead to lasting pathological consequences when the balance between bacteriostatic and bactericidal activities results in the elimination of bacterial species.^[^ ^20^, ^21^, ^22^ ^]^ To address this clinical challenge, we previously synthesized metronidazole‐fluorouracil nanoparticles (MTI‐FDU), which could effectively clear intratumoral bacteria and remodel the immune microenvironment without disrupting the balance of the intestinal microbiota, demonstrating promising efficacy in CRC chemotherapy.^[^ ^23^ ^]^

**Scheme 1:**
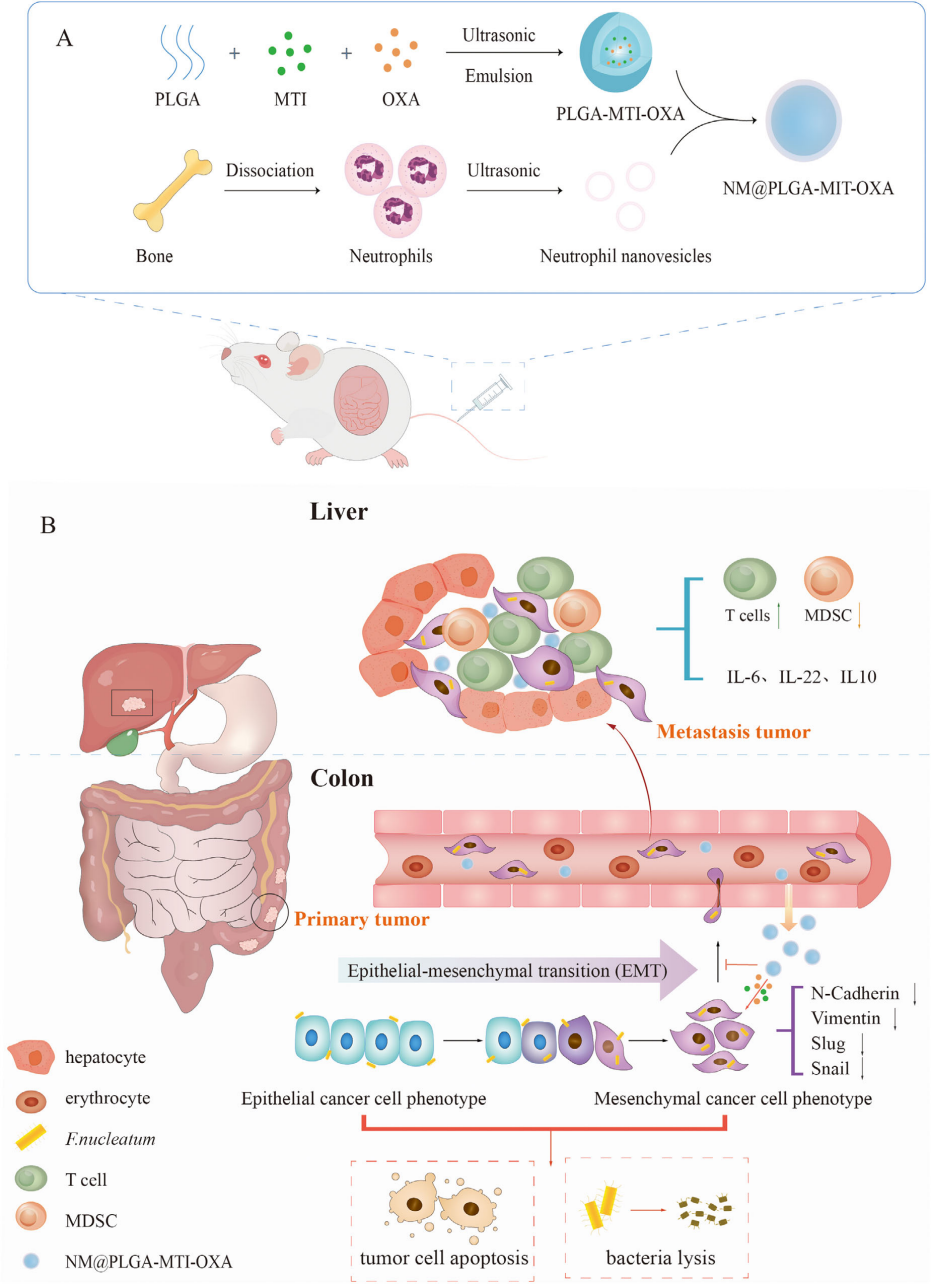
Schematic illustration of the study. A) Synthetic process of neutrophil‐mimicking nanoparticles NM@PLGA‐MTI‐OXA. B) NM@PLGA‐MTI‐OXA remodeled the tumor immune microenvironment and reversed the *Fn*‐mediated EMT process to enhance chemotherapy on liver metastasis of colorectal cancer by depleting intratumoral *Fn*.

To further investigate the efficacy of intracellular *Fn* clearance in treating CRC liver metastasis, we developed PLGA nanoparticles encapsulating MTI and OXA (PLGA‐MTI‐OXA). These nanoparticles were subsequently coated with neutrophil‐derived membrane vesicles (**Scheme** **1**A) to enhance the targeting of inflammatory colorectal tumor sites and cellular uptake by tumor cells. These neutrophil‐mimetic nanoparticles effectively reduced both surface and intracellular *Fn* in colorectal cancer cells, remodeling the immunosuppressive tumor microenvironment and reversing the epithelial‐mesenchymal transition (EMT) of tumor cells. This approach effectively inhibited CRC progression and liver metastasis without disrupting the intestinal microbiota (Scheme 1B).

## 2 Results

### 2.1 *Fn* was Highly Enriched in CRC and Liver Metastases

**Figure 1:**
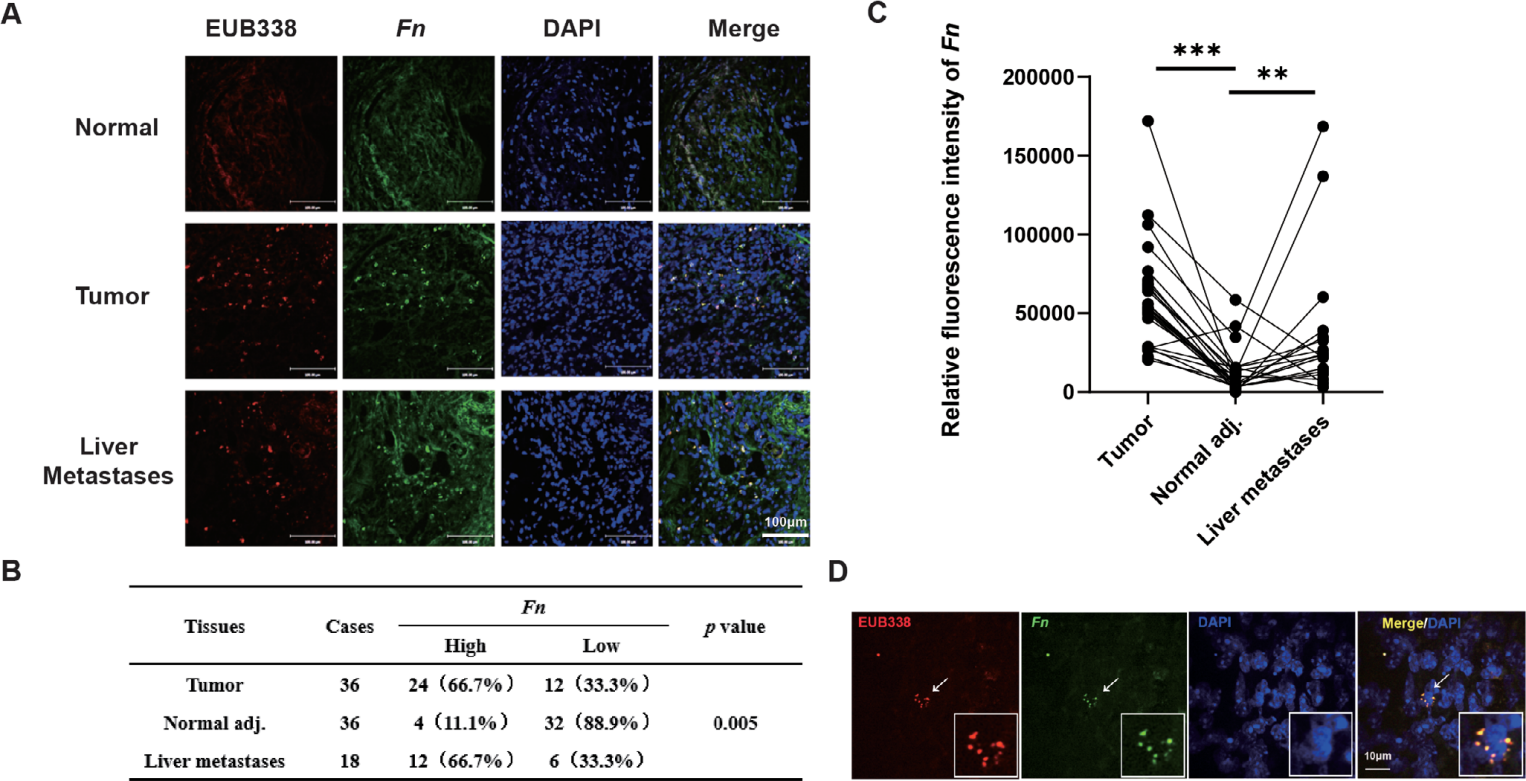
*Fn* was highly enriched in CRC and liver metastases A) Representative images of human colorectal cancer, matched adjacent nontumor tissues, and liver metastases tissues stained with *Fn*‐specific probe FUS664 (green) and universal bacterial probe EUB338 (red); cell nucleoid was stained with DAPI (blue). Scale bar = 100 µm B) Quantitative analysis of *Fn* FISH plaque. C) Quantitative analysis of an *Fn* FISH fluorescence. D) Representative images of human colorectal cancer cells stained with *Fn*‐specific probe FUS664 (green) and universal bacterial probe EUB338 (red); the cell nucleoid was stained with DAPI (blue). Scale bar = 10 µm. ^*^ *p* < 0.05; ^**^ *p* < 0.01; ^***^ *p* < 0.001.

In order to evaluate the *Fn* load in CRC tissues and liver metastases, tumor samples, matched adjacent normal tissues from 36 patients, and liver metastasis tissues from 18 of these patients were collected. *Fn* enrichment in CRC samples was analyzed using fluorescence in situ hybridization (FISH). Compared with adjacent tissues, *Fn* (FUS664 green probe) was significantly enriched in tumor tissues and liver metastases (*p* < 0.01) (**Figure** **1**A,B). Specifically, *Fn* infiltration was lower in 35 adjacent tissues compared with CRC tissues, and lower in 15 adjacent tissues compared with liver metastases (Figure 1C). Statistical analysis demonstrated a significant association between high *Fn* levels and metastasis and stage, but not with age, sex, depth of invasion and lymph node stage (Table S1, Supporting Information). Previous studies have demonstrated the presence of intratumoral bacteria within tumor cells, and *Fn* can colonize and enter tumor cells. To further determine *Fn* localization within tumors, *Fn* was labeled with Syto, CT26 cell membranes with Dil, and cell nuclei with Hoechst. Following co‐incubation of *Fn* with CT26 cells and subsequent washing, *Fn* co‐localization with the cells was observed, indicating potential adherence to the cell surface or internalization (Figure S1, Supporting Information). In clinical samples, *Fn* was enriched outside the nucleus and within the cytoplasmic matrix, suggesting *Fn* invasion and intracellular location within tumor cells (Figure 1D).

### 2.2 Characterization and Tumor Targeting of Neutrophil‐Mimicking Nanoparticles

**Figure 2:**
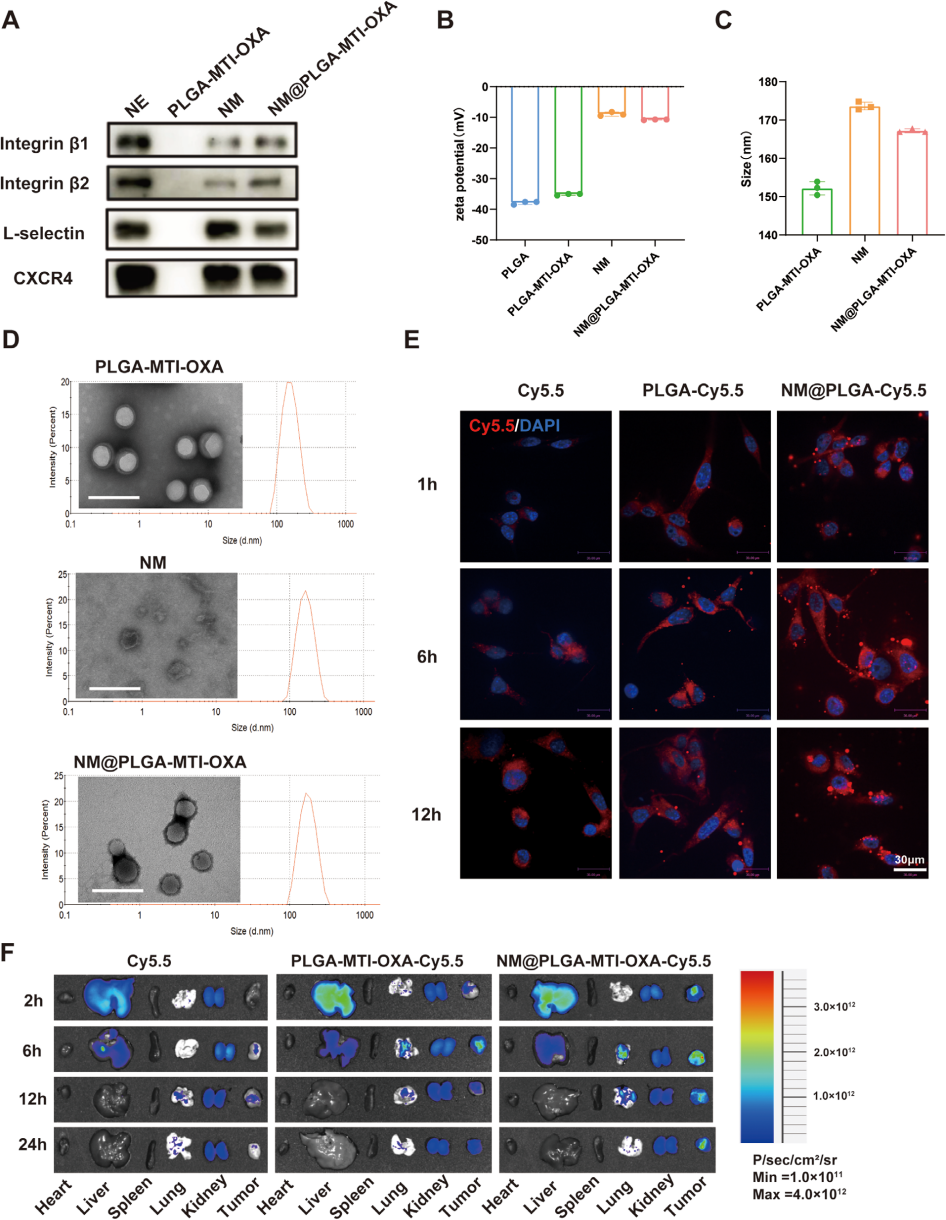
Characterization and tumor targeting of neutrophil‐mimicking nanoparticles. A) Western‐blot identification and comparison of membrane‐associated adhesive proteins, including L‐selectin, β1 integrin, β2 integrin CXCR4. B) Zeta potential C) Particle size. D)TEM image. Scale bar = 200 µm E) CLSM images of CT26 cells treated with Cy5.5, PLGA‐Cy5.5, and NM@PLGA‐Cy5.5 for 1, 6, and 12h. Cell nuclei were stained with DAPI. Scale bar = 30 µm. F) Representative images of vital organs and tumor after 2, 6, 12, and 24h post‐injection of Cy5.5, PLGA‐MTI‐OXA‐Cy5.5, and NM@ PLGA‐MTI‐OXA‐Cy5.5.

Neutrophils exhibit an inherent tendency to migrate toward sites of inflammation. To investigate the effect of *Fn* on the expression of adhesion molecules mediating neutrophil binding on colorectal cancer cells, we collected colorectal tissue samples from a spontaneous model of AOM/DSS‐induced CRC (AD model), an AD/*Fn* model, and normal mice. We observed that the expression of VCAM1, CD44, and ICAM1 was upregulated in whole intestinal tissue from the AD model compared to normal mice. Furthermore, *Fn* gavage promoted the expression of these adhesion molecules (Figure S2, Supporting Information).^[^ ^24^ ^]^ Based on the characteristics of *Fn* infiltration in colorectal cancer tissues and liver metastases, a PLGA‐coated metronidazole and oxaliplatin formulation (PLGA‐MTI‐OXA) was designed. These nanoparticles were then coated with neutrophil membrane vesicles derived from murine bone marrow. Neutrophils were isolated using a Percoll gradient centrifugation method, as previously described for murine neutrophil isolation with high purity and minimal impact on bioactivity (Figure S3A, Supporting Information). To confirm the successful translocation of neutrophil membrane proteins onto the NM@PLGA‐MTI‐OXA surface, protein content was quantified using a Coomassie blue assay (Figure S3B, Supporting Information). Membrane isolation resulted in a distinct protein profile compared to whole neutrophils. Western blot analysis demonstrated the enrichment of key adhesion proteins—including integrin β1, integrin β2, L‐selectin, and CXCR4 on NM@PLGA‐MTI‐OXA, thus supporting the neutrophil‐mimetic properties of the construct (**Figure** **2**A). DiO staining of neutrophil membranes confirmed the successful camouflage of NM@PLGA‐Cy5.5 with the membrane coating (Figure S3C, Supporting Information). The ζ‐potential of NM@PLGA‐MTI‐OXA increased by ≈30 mV compared to bare PLGA‐MTI‐OXA, approaching the level of the membrane vesicles (Figure 2B). Transmission electron microscopy (TEM) and dynamic light scattering (DLS) revealed a core‐shell structure for NM@PLGA‐MTI‐OXA, with a mean size of 167.2 ± 0.5 nm (Figure 2C,D). The hydrodynamic diameter of NM@PLGA‐MTI‐OXA remained stable for one week in various solutions, including water, phosphate‐buffered saline (PBS), and 10% fetal bovine serum (FBS) (Figure S4, Supporting Information). The successful loading of MTI was confirmed by UV–vis spectroscopy, and OXA loading was quantified by high‐performance liquid chromatography (HPLC). The entrapment efficiencies for MTI and OXA in PLGA‐MTI‐OXA were ≈21% and 65%, respectively.

Cellular uptake in vitro and biodistribution in vivo were subsequently investigated. CT26 cells were treated with equivalent amounts of Cy5.5, PLGA‐Cy5.5, or NM@PLGA‐Cy5.5 and then analyzed by confocal laser scanning microscopy (CLSM). With increasing incubation time, the fluorescence intensity of the NM@PLGA‐Cy5.5 group in CT26 cells increased significantly compared to the Cy5.5 and PLGA‐Cy5.5 groups (Figure 2E; Figure S5, Supporting Information). To further investigate the effect of *Fn* on the uptake of nanoparticles by tumor cells, we treated *Fn*‐infected CT26 with NM@PLGA‐Cy5.5 for 6 h, then detected the expression of adhesion molecules via immunofluorescence. The results showed that compared to the control group, the expression of VCAM1(Figure S6A, Supporting Information), CD44(Figure S6B, Supporting Information), and ICAM1(Figure S6C, Supporting Information) was upregulated in *Fn*‐infected CT26 cells, and more NM@PLGA‐Cy5.5 particles were observed which suggested that the high expression of adhesion molecules facilitates the adhesion of NM@PLGA‐Cy5.5 to tumor cells, ensuring efficient uptake of NM@PLGA‐Cy5.5 by the tumor cells. Additionally, we treated *Fn*‐infected CT26 cells with equal amounts of PLGA‐Cy5.5 and NM@PLGA‐Cy5.5, then detected the localization of adhesion molecules and nanoparticles via immunofluorescence. We observed that red fluorescent particles in the NM@PLGA‐Cy5.5 group colocalized with adhesion molecules were significantly more abundant than in the PLGA‐Cy5.5 group, especially on the surface of cells (Figure S7A–C, Supporting Information). The result indicated that the neutrophil membrane‐coated effectively enhanced the binding of nanoparticles and adhesion molecules so that NM@PLGA‐Cy5.5 nanoparticles tightly adhered to the surface of tumor cells after multiple rounds of washing in the immunofluorescence assay.

Further, the results of the lysosome escape assay indicated that both PLGA‐Cy5.5 and NM@PLGA‐Cy5.5 were efficiently taken up by tumor cells (Figure S8, Supporting Information). It was found that NM@PLGA‐Cy5.5 group presented more intense signals than PLGA‐Cy5.5, which indicated that the covered membrane components promoted the uptake efficiency, which reinforced the related conclusion obtained from the above assays. What's more, there were obviously more red signals existing in NM@PLGA‐Cy5.5 incubated cells in addition to yellow fluorescence after 1h and 6h of treatment compared to PLGA‐Cy5.5 group, suggesting that except for a part of NM@ PLGA‐Cy5.5 dragged in endosome/lysosome system there were a large part escaped into cytoplasm which indicated that the encapsulating of neutrophil membrane might further improved the capacity of nanoparticles to escape from endosome/lysosome. After 12 h of treatment, lysosome escape consistently appeared in the PLGA‐Cy5.5 and NM@ PLGA‐Cy5.5 groups.

In addition, in vivo optical imaging was conducted to investigate the accumulation of NM@PLGA‐Cy5.5 in the intestinal tract and other tissues. NM@PLGA‐Cy5.5, PLGA‐Cy5.5, and free Cy5.5 were administered intravenously to a colitis/*Fn* model. The Cy5.5 fluorescence signal was recorded using a multimode optical imaging system at 6 h post‐injection. The fluorescence intensity in colon tissue of the NM@PLGA‐Cy5.5 group was higher than that of the PLGA‐Cy5.5 and free Cy5.5 groups (Figure S9, Supporting Information). To determine whether nanoparticles could target tumor sites, in vivo optical imaging was performed. NM@PLGA‐MTI‐OXA‐Cy5.5, PLGA‐MTI‐OXA‐Cy5.5, and free Cy5.5 were administered intravenously to tumor‐bearing mice. The Cy5.5 fluorescence signal was recorded using a multimode optical imaging system at 2, 6, 12, and 24h post‐injection. The fluorescence intensity in tumor tissue of the Cy5.5 group decreased from 2 h post‐injection, falling below 50% within 24 h. In contrast, the fluorescence intensity in tumor tissue of the NM@PLGA‐MTI‐OXA‐Cy5.5 group remained high at both time points (Figure 2F; Figure S10, Supporting Information). To further investigate the distribution of MTI and OXA in plasma and tumor tissue, their concentrations were measured 6 h after injection of MTI+OXA, PLGA‐MTI‐OXA, and NM@PLGA‐MTI‐OXA. The results demonstrated that the concentrations of MTI and OXA in the tumor tissue of the NM@PLGA‐MTI‐OXA group were higher than those of the MTI+OXA and PLGA‐MTI‐OXA groups. However, no significant differences in plasma concentrations of MTI and OXA were observed among the treatment groups (Figure S11, Supporting Information). Overall, these results confirmed that NM@PLGA‐MTI‐OXA could effectively accumulate at tumor sites.

### 2.3 Bactericidal and Tumoricidal Effect of Neutrophil‐Mimicking Nanoparticles in vitro

**Figure 3:**
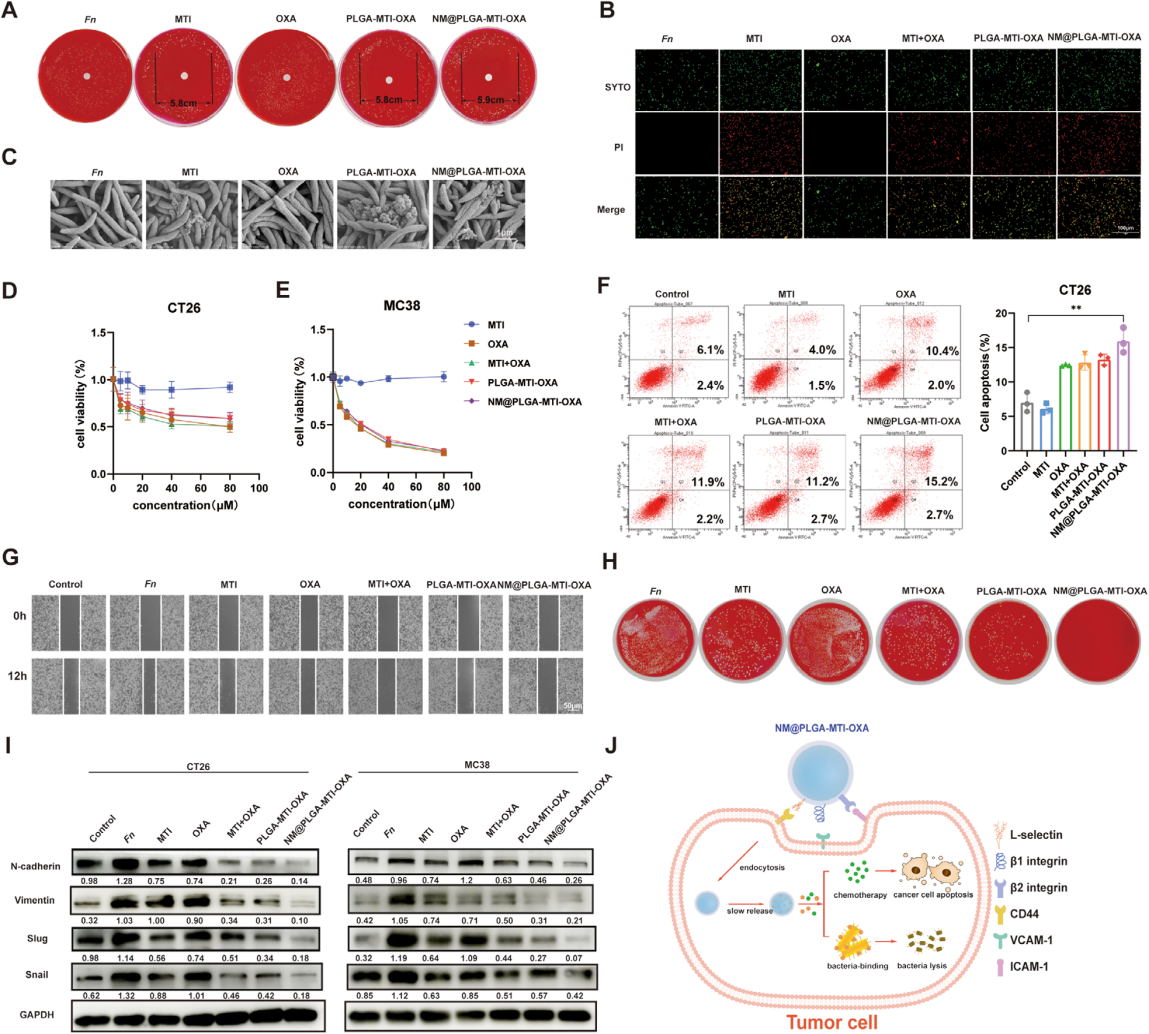
Bactericidal and tumor cell killing effect of neutrophil‐mimicking nanoparticles in vitro. A) Antibacterial efficiency of different drugs after 48h by paper diffusion assay. B) CLSM images of live/dead *Fn* stained with SYTO9/PI treated with different drugs for 6h as before. Scale bar = 100 µm. C) SEM micrographs of *Fn* treated with PBS (control), MTI, OXA, MTI+OXA, PLGA‐MTI‐OXA, and NM@ PLGA‐MTI‐OXA for 6 h. Scale bar = 1 µm. D,E) Cell viability after treatment of CT26 cells and MC38 with different concentrations of different drugs for 24 h. F) Flow cytometry analysis with Annexin V/PI staining evaluating the percentages of apoptotic cells among different drug treatment groups. G) Wound healing assay of CT26 cells treated with different drugs for 12 h. H) Colony plate images after cell lysis, cultured with *Fn* and then treated with PBS (control), MTI, OXA, MTI+OXA, PLGA‐MTI‐OXA, and NM@ PLGA‐MTI‐OXA at 6 h. I) The expression of EMT related protein, including Vimentin, Slug, and Snail, after being infected with *Fn* for 2h and treated with different drugs for 24 h. J) The schematic diagram illustrates the mode of action wherein Neutrophil‐Mimicking nanoparticles conjugate with cell membrane surface markers to enter cells, releasing antibiotics to kill bacteria and simultaneously releasing oxaliplatin to damage tumor cells. ^*^ *p* < 0.05; ^**^ *p* < 0.01; ^***^ *p* < 0.001.

To assess the antibacterial activity of NM@PLGA‐MTI‐OXA, a paper diffusion drug sensitivity test was performed. The resulting inhibition zone diameters were as follows: MTI, 5.8 cm; PLGA‐MTI‐OXA, 5.8 cm; and NM@PLGA‐MTI‐OXA, 5.9 cm. OXA exhibited no inhibitory activity (**Figure** **3**A; Figure S12, Supporting Information). Live/dead staining of the bacteria revealed consistent outcomes, demonstrating the absence of viable *Fn* after treatment with MTI, PLGA‐MTI‐OXA, and NM@PLGA‐MTI‐OXA, as evidenced by the predominant red fluorescence observed in the CLSM images. In contrast, OXA exhibited no inhibitory effect on *Fn* viability (Figure 3B). Scanning electron microscopy (SEM) images revealed comparable damage to the *Fn* cell membranes in the MTI, MTI+OXA, PLGA‐MTI‐OXA, and NM@PLGA‐MTI‐OXA groups compared to the control (PBS) and OXA treatment groups (Figure 3C). These findings collectively indicated that the antibacterial effect of NM@PLGA‐MTI‐OXA was equivalent to that of the MTI drug alone. These results demonstrated that NM@PLGA‐MTI‐OXA could effectively kill *Fn*, providing a foundation for the development of therapeutic interventions targeting intratumoral bacterial populations and disrupting pathogenic tumor symbionts.

To investigate the effect of NM@PLGA‐MTI‐OXA on CRC cells, an in vitro cytotoxicity study was performed. MTI did not significantly affect CRC cell viability, whereas PLGA‐MTI‐OXA and NM@PLGA‐MTI‐OXA exhibited similar dose‐dependent inhibition of CT26 and MC38 cells, comparable to the OXA and MTI+OXA groups (Figure 3D,E). Subsequently, in vitro apoptosis of colorectal tumor cells was assessed following nanodrug treatment. Apoptosis induced by PLGA‐MTI‐OXA, NM@PLGA‐MTI‐OXA, and OXA was drug concentration‐dependent, indicating that nanosizing retained the chemotherapeutic agents' ability to effectively induce apoptosis in CRC cells (Figure 3F; Figure S13A, Supporting Information). Given that *Fn* promotes CRC migration, a wound‐healing assay was conducted to measure CT26 and MC38 cell migration in the presence of *Fn* with different drug treatments. NM@PLGA‐MTI‐OXA demonstrated superior inhibition of tumor cell migration compared to MTI, MTI+OXA, and PLGA‐MTI‐OXA (Figure 3G; Figure S13B–D, Supporting Information). These results suggested that neutrophil membrane‐coated nanoparticles could enhance cellular uptake, effectively eradicating bacteria within tumor cells. Therefore, following 2 h of co‐incubation of CT26 with *Fn*, drugs were added for 4 h. Subsequently, cells were lysed, plated onto Columbia blood agar plates, and anaerobically cultured for 48 h to observe bacterial colonies. MTI, MTI+OXA, and PLGA‐MTI‐OXA groups still harbored bacteria, forming multiple *Fn* colonies. In contrast, the NM@PLGA‐MTI‐OXA group showed no bacterial colony formation, demonstrating its ability to effectively eradicate *Fn*, particularly within tumor cells (Figure 3H; Figure S14, Supporting Information). EMT, a key molecular step in colorectal cancer distant metastasis, is typically accompanied by the expression of mesenchymal markers such as vimentin, Slug, Snail, and N‐cadherin.^[^ ^25^ ^]^ Mechanistically, the impact of NM@PLGA‐MTI‐OXA on the expression of EMT‐related proteins in tumor cells infiltrated by *Fn* was evaluated. Results showed increased expression of N‐cadherin, vimentin, Slug, and Snail in tumor cells treated with *Fn*. Conversely, treatments with MTI, MTI+OXA, PLGA‐MTI‐OXA, and NM@PLGA‐MTI‐OXA reversed the elevated expression of these EMT‐related proteins, with NM@PLGA‐MTI‐OXA exhibiting the most pronounced inhibitory effect, likely attributable to its effective eradication of *Fn* within the tumor cells (Figure 3I; Figure S15, Supporting Information). To further investigate the effect of *Fn* on tumor cell migration and distant metastasis, CT26 and HCT116 cells were co‐incubated with *Fn*. Cytoskeletal remodeling was observed in these cells after 6 h, accompanied by morphological changes indicative of *Fn*‐mediated promotion of mesenchymal‐like cell morphology, thereby facilitating distant metastasis of tumor cells (Figure S16A,B, Supporting Information).

Overall, combined with the results in Figures S6 and S7 (Supporting Information), NM@PLGA‐MTI‐OXA can be specifically internalized by tumor cells via surface‐coated neutrophil membranes, which could bind to the adhesion molecule on the surface of tumor cells, facilitating the intracellular release of MTI and OXA. This process effectively eliminates intracellular bacteria and reverses the promoting effects of *Fn* on EMT‐related protein expression and cytoskeletal remodeling in tumor cells, thereby enhancing the anti‐tumor efficacy of OXA (Figure 3J).

### 2.4 Neutrophil‐Mimicking Nanoparticles can Effectively Impede the Progression of Spontaneous and Axillary Colorectal Cancer

**Figure 4:**
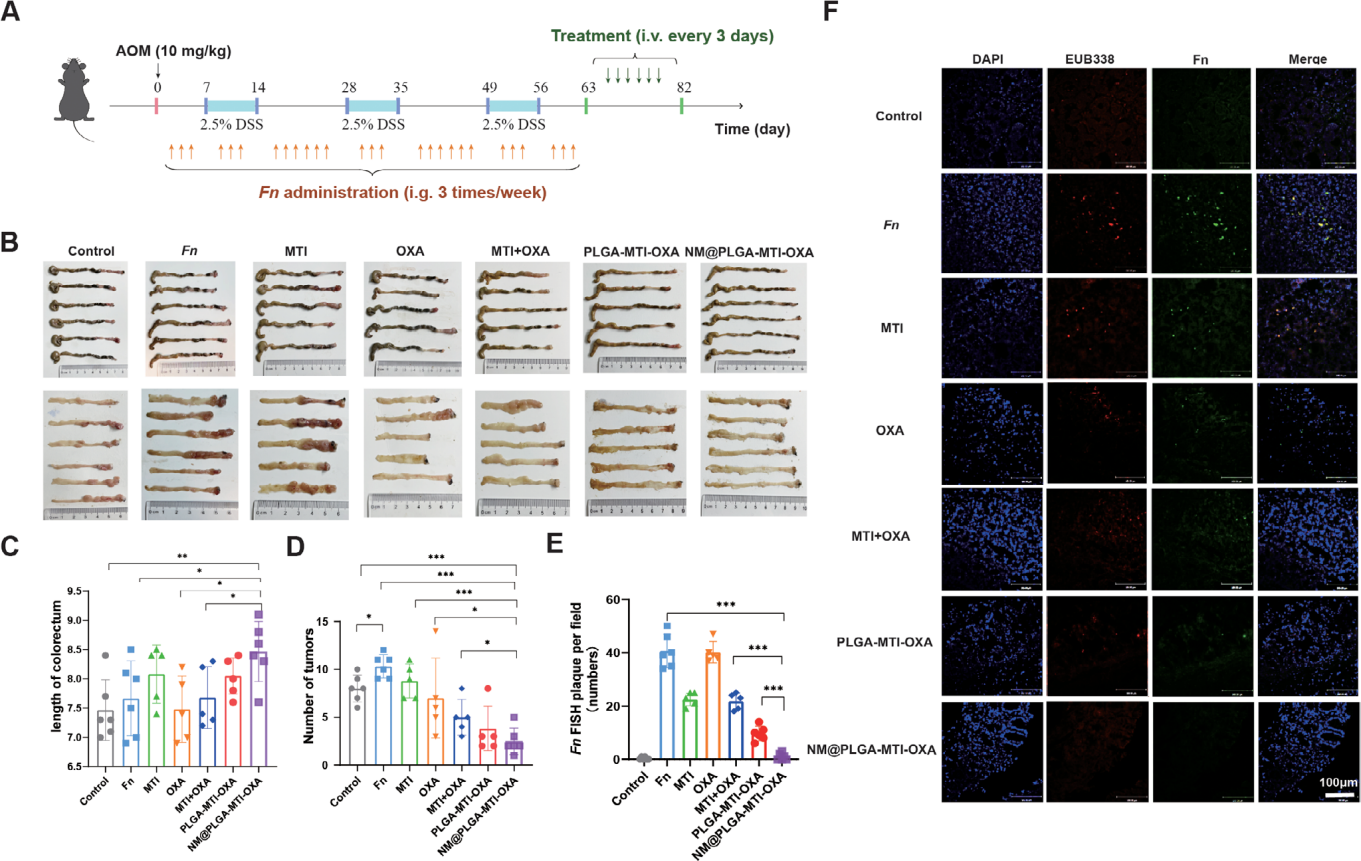
Neutrophil‐mimicking nanoparticles can effectively delay the progression of an AD/*Fn* CRC spontaneous model. A) Timeline of the spontaneous model of CRC in vivo treatment. B) General view of the mouse colorectum and anatomical atlas of the interior colorectum after dissection. C) Length of the mouse colorectum. D) Statistical graph of the number of tumor nodules in the mouse colorectum. E) Quantitative analysis of *Fn* FISH plaques in F. F) Photographs of CLSM taken by FISH to detect *Fn* infiltration in colorectal sites of tumors, blue: DAPI‐labeled nuclei, red: CY5‐EUB338 universal bacterial probes, green: FAM‐FUS664 probe, scale bar = 100 µm. (*n* = 5 or 6) ^*^ *p* < 0.05; ^**^ *p* < 0.01; ^***^ *p* < 0.001.

The AOM/DSS‐induced CRC model represents a spontaneous model of transformation from colorectal inflammation to cancer. To evaluate the therapeutic efficacy of NM@PLGA‐MTI‐OXA targeting both tumor bacteria and tumor cells, the AOM/DSS/*Fn* CRC model was employed. CRC was induced in C57BL/6 mice using AOM/DSS (**Figure** **4**A), with each mouse receiving *Fn* via oral gavage throughout the induction process to facilitate bacterial infiltration into tumors after intestinal colonization. Mice were randomly assigned to treatment groups and received intravenous injections via the tail vein: control group (PBS), MTI, OXA, MTI+OXA (combination), PLGA‐MTI‐OXA, and NM@PLGA‐MTI‐OXA. Treatments were administered three times per week for three weeks. At the experiment's conclusion, colons were dissected, and colon length and the number of visible tumor nodules were measured and recorded. Mouse body weight was monitored throughout the treatment period.

**Figure 5:**
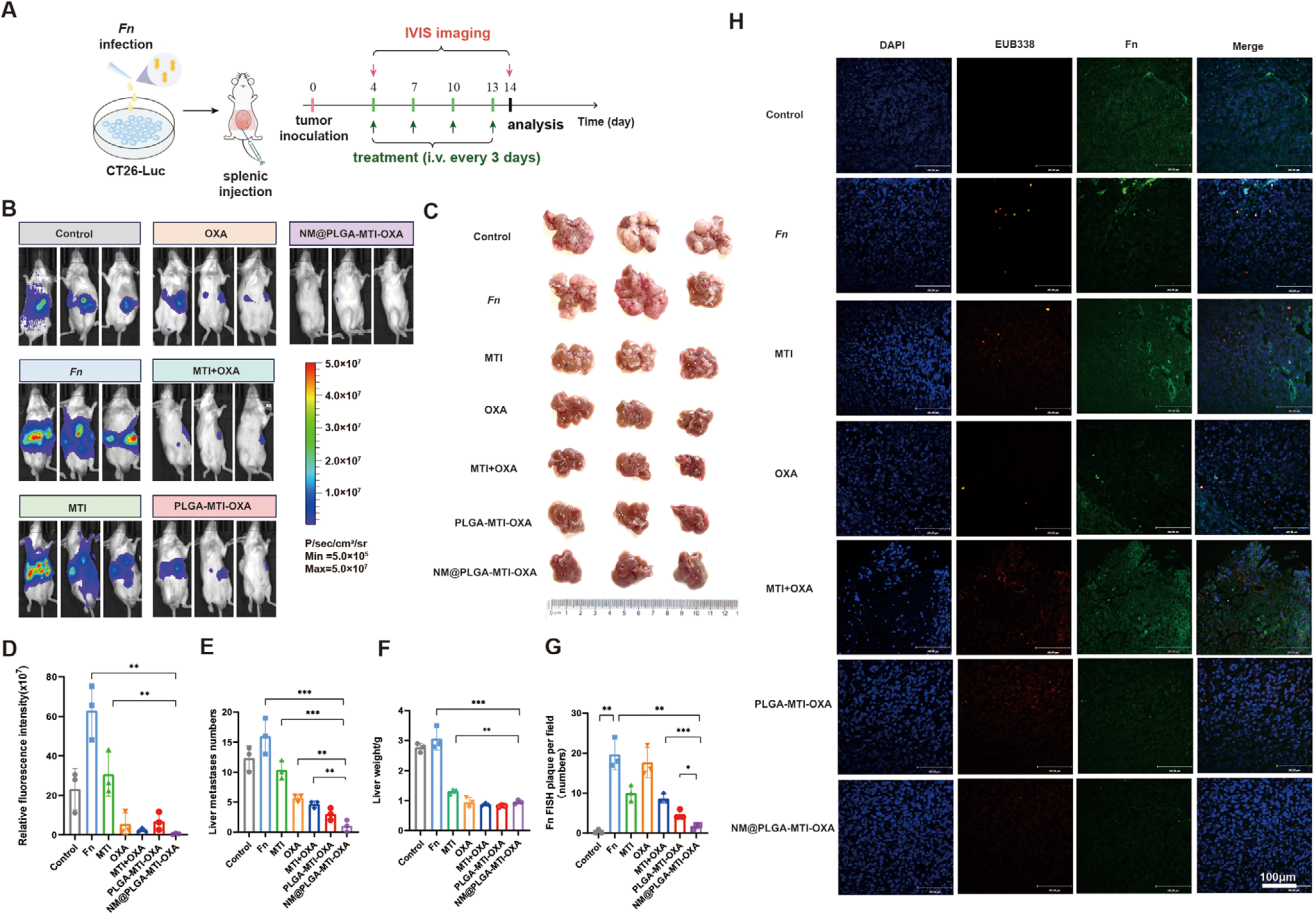
Neutrophil‐mimicking nanoparticles can effectively delay the progression of *Fn*‐infected liver metastasis model. A) Timeline of *Fn*‐infected liver metastasis model. B) IVIS images of bioluminescence signal of the luciferase assay for each mouse at Day 14. C) General view of representative dissected liver at day 14. D) Quantitative analysis of fluorescence in (B). E) Statistical graph of the number of tumor nodules in liver metastasis. F) The liver weight in groups treated with different drugs. G) Quantitative analysis of *Fn* FISH plaques in H. H) Photographs of CLSM taken by FISH to detect *Fn* infiltration in liver metastasis sites, blue: DAPI‐labeled nuclei, red: CY5‐EUB338 universal bacterial probes, green: FAM‐FUS664 probe, scale bar = 100 µm. (*n* = 3) ^*^ *p* < 0.05; ^**^ *p* < 0.01; ^***^ *p* < 0.001.

Colorectal length in mice serves as an indicator of intestinal inflammation and indirectly reflects drug therapeutic efficacy. During the AOM/DSS‐induced ulcerative colitis to colon cancer transition, a notable decrease in colorectal length was observed. However, NM@PLGA‐MTI‐OXA treatment reversed this effect, preventing intestinal shortening during tumorigenesis (Figure 4B,C). Furthermore, *Fn* gavage increased the number and size of tumor nodules, indicating that *Fn* infiltration could promote colorectal cancer development. NM@PLGA‐MTI‐OXA administration significantly reduced the number of colorectal tumor nodules, with an inhibition rate reaching 75.8% compared to *Fn* group, indicating substantial therapeutic efficacy. (**Figure** **5**D). Body weight (Figure S17A, Supporting Information), hematoxylin and eosin (H&E) analysis of major organs (Figure S18A, Supporting Information), and blood biochemistry results for liver and kidney function (Figure S18B–D, Supporting Information) indicated that NM@PLGA‐MTI‐OXA exhibited minimal toxicity and side effects. In summary, NM@PLGA‐MTI‐OXA exhibited effective therapeutic outcomes against colorectal cancer with minimal toxicity. *Fn* infiltration in tumors was detected using FISH. Abundant *Fn* infiltration was observed in tumors of mice treated with *Fn*, MTI, OXA, MTI+OXA, and PLGA‐MTI‐OXA, with minimal variation in infiltration levels, suggesting limited efficacy of free drugs against intratumoral bacteria. In contrast, *Fn* infiltration was scarcely observed in the NM@PLGA‐MTI‐OXA group (Figure 4E,F). These results demonstrated that the NM@PLGA‐MTI‐OXA nanodrug could target intratumoral *Fn* while exerting potent tumor‐inhibiting effects.

To more accurately and directly assess the impact of NM@PLGA‐MTI‐OXA nanomedicine on CRC, a CRC axillary MC38/*Fn* tumor model was established (Figure S19A, Supporting Information). *Fn* was injected peritumorally to infiltrate the tumor. Analysis of the tumor growth curve and tumor images revealed that tumors in the NM@PLGA‐MTI‐OXA nanodrug group were significantly smaller and weight loss compared to the control and other treatment groups (Figure S19B–E, Supporting Information). The tumor growth inhibition rate of it was achieved 75.7% relative to the *Fn* group, which demonstrated the nanodrug's effectiveness in therapeutic outcome. Furthermore, almost all infiltrated *Fn* within the tumor was eliminated in the NM@PLGA‐MTI‐OXA group, indicating that the nanomedicine could target the tumor site and exert antibacterial effects (Figure S19F,G, Supporting Information). H&E staining of major organs (Figure S20A, Supporting Information) and blood biochemical analyses assessing liver and kidney function (Figure S20B–E, Supporting Information) collectively suggested that NM@PLGA‐MTI‐OXA did not exhibit detectable drug toxicity or adverse effects in vivo.

### 2.5 Neutrophil‐Mimicking Nanoparticles Can Effectively Delay the Progression of the *Fn*‐Infected Liver Metastasis Model

Liver metastasis, affecting up to 70% of patients with CRC, represents the most common form of distant metastasis. To investigate the effects of NM@PLGA‐MTI‐OXA on CRC liver metastasis, a *Fn*‐infected liver metastasis model was established (Figure 5A). Infected CT26‐luc cells were inoculated via the portal vein by splenic injection, resulting in uniform liver metastasis, which was more severe compared to that observed with uninfected cells (Figure S21, Supporting Information). Treatment with OXA, MTI+OXA, PLGA‐MTI‐OXA, and NM@PLGA‐MTI‐OXA inhibited the development of liver metastasis, as quantified by in vivo luciferase imaging and analysis of sectioned liver tissue (Figure 5B,C). NM@PLGA‐MTI‐OXA significantly reduced tumor burden compared to the PBS control, as quantified by luciferase signals (Figure 5D). Due to the increased number of liver metastases in the *Fn* group, liver weight was significantly higher compared to other treatment groups; NM@PLGA‐MTI‐OXA treatment resulted in the most significant decrease in the number of liver metastases, with an inhibition rate reached 93.75% compared to the *Fn* group (Figure 5E). While MTI alleviated *Fn*‐induced liver metastasis, leading to a slight decrease in liver weight, the overall difference in liver weight among the other treatment groups was not significant (Figure 5F). FISH imaging for *Fn* revealed a high bacterial abundance in untreated liver metastases, which was significantly reduced following NM@PLGA‐MTI‐OXA treatment (Figure 5G,H). Changes in body weight among the treatment groups were not significant, potentially due to the relatively short treatment duration compared to the other two models (Figure S17B, Supporting Information).

### 2.6 Neutrophil‐Mimicking Nanoparticles Maintained Intestinal Flora Balance in Mice

**Figure 6:**
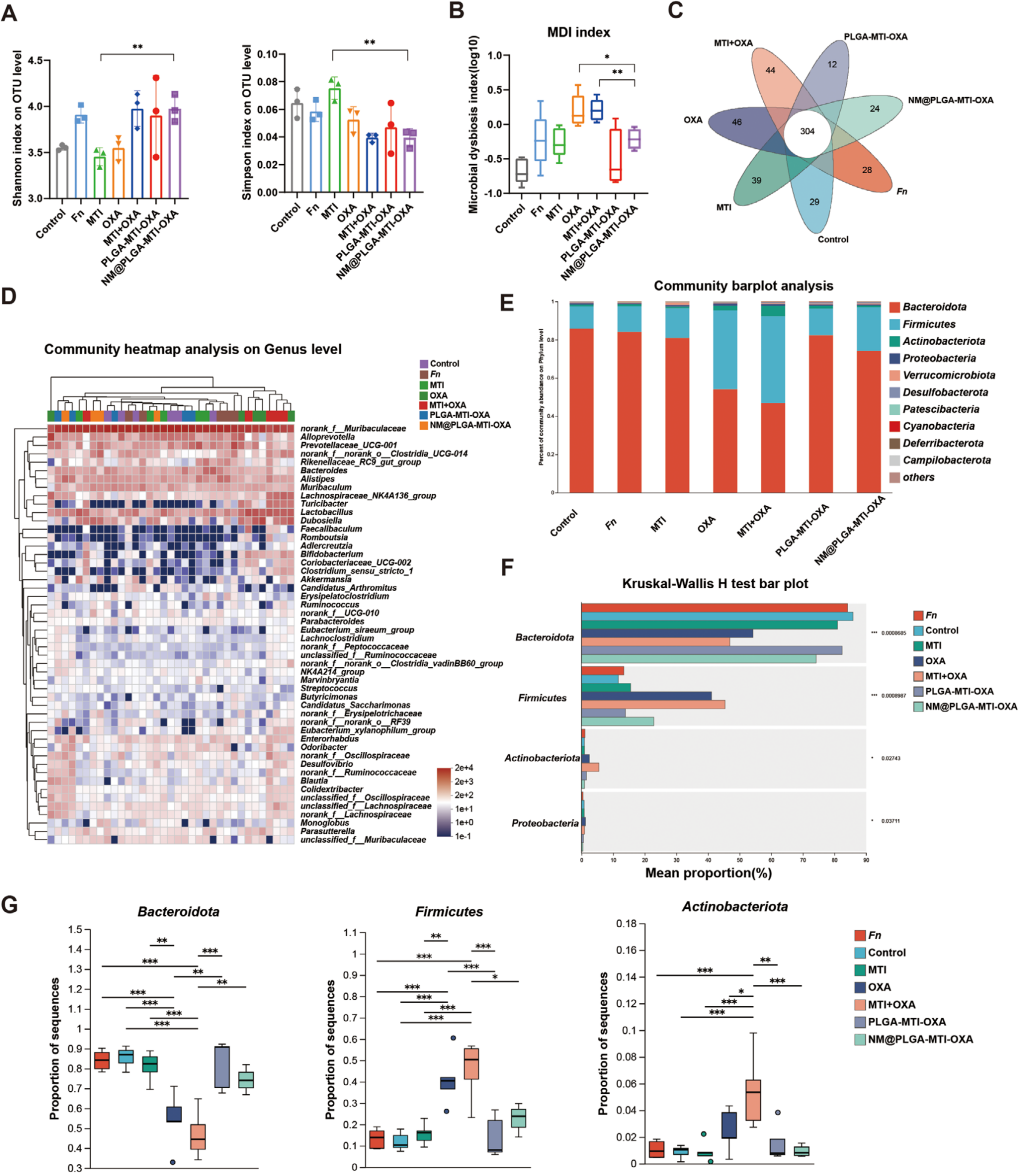
Neutrophil‐mimicking nanoparticles maintained intestinal flora balance in AD/*Fn* CRC spontaneous model. A) Analysis of alpha diversity of intestinal flora, Shannon index, and Simpson index was observed by 16S rDNA sequencing. B) Microbiota dysbiosis index (MDI) of different groups. C) Venn analysis of species in different treatment groups. D) Community heatmap analysis on the genus level was performed to explore the differences in intestinal flora composition after different drug treatments. E) Stacked bar plot of the phylum level relative abundance of bacteria communities in the indicated samples. F) Differential analysis of the top 4 fecal flora in abundance in different treatment groups at the phylum level. G) Quantitative analysis of the top 3 fecal flora in abundance in different treatment groups at the phylum level. (*n* = 3) ^*^ *p* < 0.05; ^**^ *p* < 0.01; ^***^ *p* < 0.001.

Gut dysbiosis induced by antibiotics and chemotherapeutic agents can significantly alter the composition and abundance of gut microbiota, thereby influencing tumor progression and contributing to various adverse effects. Therefore, the role of NM@PLGA‐MTI‐OXA in maintaining intestinal microbial balance was further investigated. Fecal samples were collected from mice in both the AD/*Fn* CRC spontaneous model and the MC38/*Fn* CRC tumor model for 16S rDNA sequencing. Alpha diversity was assessed using Shannon, Ace, Sobs, Chao, and Simpson indices. In the AD/*Fn* CRC spontaneous model, the MTI group exhibited significantly lower microbiota diversity and richness compared to the control group, whereas no significant changes were observed in the NM@PLGA‐MTI‐OXA group (**Figure** **6**A). Similarly, in the MC38/*Fn* model, the MTI+OXA group showed significantly lower microbiota diversity and richness than the control group, while the NM@PLGA‐MTI‐OXA group showed no significant changes, indicating a substantial impact of MTI+OXA on the intestinal microbiota in this model (Figure S22A, Supporting Information). Microbial dysbiosis index (MDI) analysis revealed gut dysbiosis in the OXA and MTI+OXA groups relative to the control group, while no dysbiosis was observed in the nanoparticle groups (Figure 6B; Figure S22B, Supporting Information). A Venn plot revealed variations in species richness across different treatment groups (Figure 6C; Figure S22D, Supporting Information). Heatmap analysis at the genus level demonstrated greater similarity in bacterial composition between the nanodrug and control groups compared to the MTI or MTI+OXA groups (Figure 6D; Figure S22C, Supporting Information). In the control group, the five most abundant genera were *Bacteroidota, Firmicutes, Actinobacteriota, Proteobacteria, and Verrucomicrobiota*. However, treatment with OXA or MTI combined with OXA significantly reduced the abundance of *Bacteroidota*, while the relative abundance of *Firmicutes* and *Actinobacteriota* increased. In the AD/*Fn* CRC spontaneous model, no significant changes were observed in the PLGA‐MTI‐OXA and NM@PLGA‐MTI‐OXA groups (Figure 6E–G), contrasting with the MC38/*Fn* axillary colorectal cancer model (Figure S22E, Supporting Information). These findings suggested that antibiotics and chemotherapeutic agents might yield detrimental effects on the gut microbiota, with chemotherapeutic agents such as OXA potentially exerting a greater impact than antibiotic treatment. Notably, NM@PLGA‐MTI‐OXA appeared to mitigate these effects. The observed differences between the two models may be attributed to the presence of enteritis throughout the experimental cycle in the AD/*Fn* CRC spontaneous model, which more closely mimics the intestinal environment of clinical colitis‐associated colorectal cancer. Conversely, the MC38/*Fn* colorectal axillary tumor model provides a clearer assessment of the direct effects of drugs on the intestines, necessitating an integrated analysis of both models.

### 2.7 Neutrophil‐Mimicking Nanoparticles Remodeled the Tumor Immune Microenvironment and Reversed the *Fn*‐Mediated EMT Process

**Figure 7:**
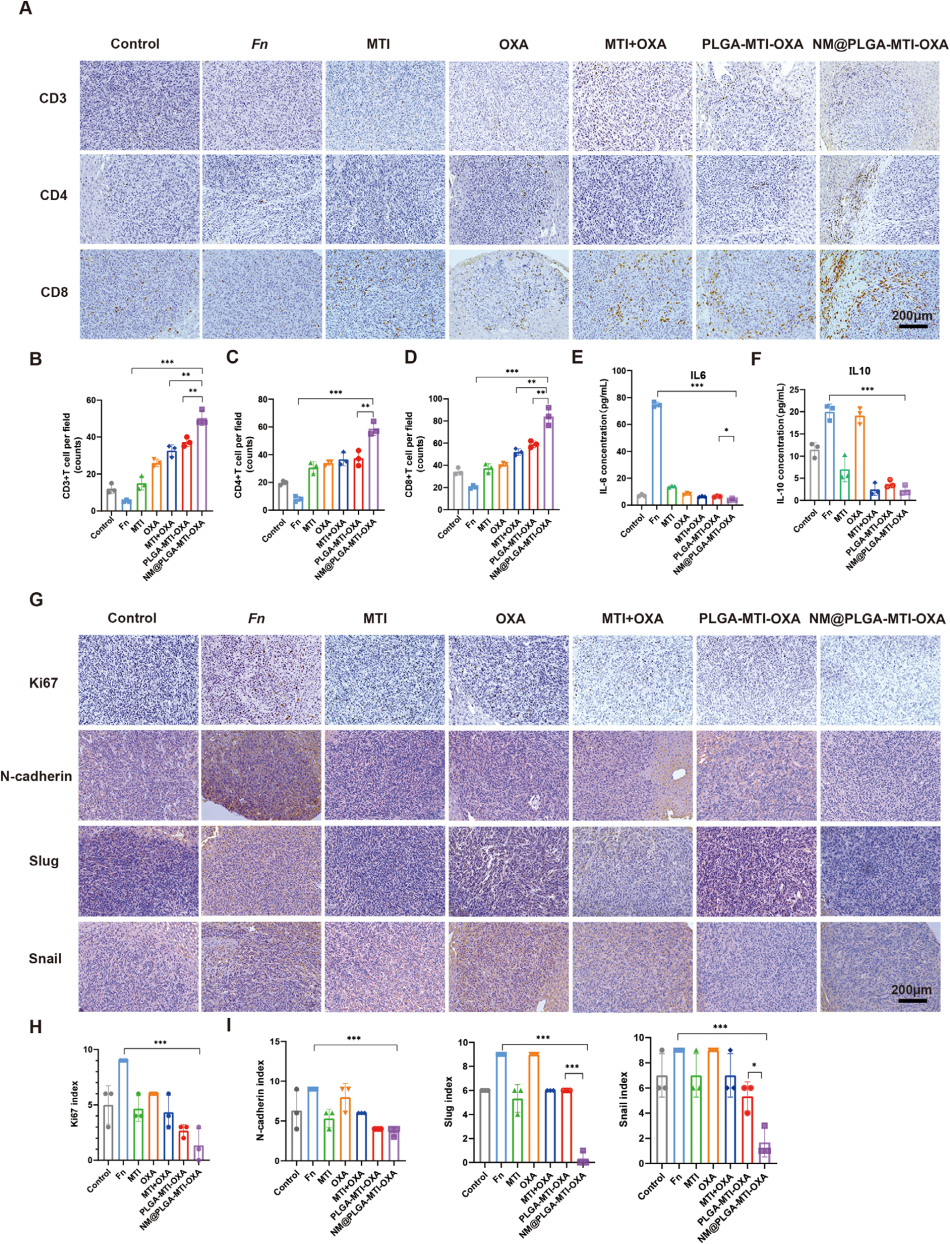
Neutrophil‐mimicking nanoparticles remodeled the tumor immune microenvironment and reversed the *Fn*‐mediated EMT process in *Fn*‐infected liver metastasis model. A) Representative IHC staining images of CD3, CD4, CD8 in *Fn*‐infected liver metastasis tissues. Scale bar = 200 µm. B,C,D) Quantitative analysis in A. E,F) Cytokine levels of IL‐6, and IL‐10 in serum were detected through ELISA among different treated groups. G) Representative IHC staining images of Ki67, N‐cadherin, slug, and snail in *Fn*‐infected liver metastasis tissues. Scale bar = 200 µm. H,I) Quantitative analysis in G. (*n* = 3) ^*^ *p* < 0.05; ^**^ *p* < 0.01; ^***^ *p* < 0.001.

Recent studies have demonstrated that the infiltration of *Fn* into tumors reduces cytotoxic CD8^+^ T cell infiltration, inhibits T cell cytotoxic function, and contributes to the formation of an immunosuppressive tumor microenvironment.^[^ ^26^, ^27^, ^28^ ^]^ To investigate the effect of NM@PLGA‐MTI‐OXA on modulating the immune microenvironment, immunohistochemical staining for CD3, CD4, and CD8 proteins was performed on tumor sections from the AD/*Fn* and *Fn*‐infected liver metastasis models, as well as the MC38/*Fn* colon axillary tumor model. Following NM@PLGA‐MTI‐OXA treatment, increased infiltration of CD3^+^, CD4^+^, and CD8^+^ T cells into intestinal tumors was observed (**Figure** **7**A–D; Figures S23A–D and S24A–D, Supporting Information). Flow cytometry analysis showed an increase in CD4^+^ and CD8^+^ T cells in the peripheral blood of the *Fn*‐infected liver metastasis after NM@PLGA‐MTI‐OXA treatment which was ≈1.85‐fold and ≈2.56‐fold of that in *Fn* group (Figure S25A–C, Supporting Information), and a significant reduction on ≈2.53‐fold in myeloid‐derived suppressor cell (MDSC) levels (Figure S25A,D, Supporting Information). In AD/*Fn* models, the CD4^+^ and CD8^+^ T cells in NM@PLGA‐MTI‐OXA group were ≈2.43‐fold and ≈2.69‐fold of that in *Fn* group, suggesting an up‐regulation of tumor immunity (Figure S25E–G, Supporting Information). Plasma levels of inflammation‐related cytokines, including IL‐6, IL‐10, and IL‐22, were also measured post‐treatment. In the *Fn*‐infected liver metastasis model, IL‐6 and IL‐10 levels were upregulated in the *Fn* group but downregulated after treatment with antibiotics and chemotherapeutic agents, particularly in the NM@PLGA‐MTI‐OXA group, which exhibited the lowest expression levels (Figure 7E,F). In the AD/*Fn* model, IL‐6 and IL‐22 levels were upregulated after *Fn* gavage but downregulated following treatment with antibiotics and chemotherapeutic agents (Figure S23E,F, Supporting Information). These findings indicated that the NM@PLGA‐MTI‐OXA nanodrug reverses the *Fn*‐mediated immunosuppressive microenvironment, increases cytotoxic CD8^+^ and CD4^+^ T cell infiltration, effectively reduces inflammatory cytokine expression in the colorectal cancer mouse model, and enhances the efficacy of the chemotherapeutic agent oxaliplatin after clearing intratumoral bacteria.

Additionally, Ki67 expression was assessed in each group. *Fn* infiltration was found to promote Ki67 expression, whereas NM@PLGA‐MTI‐OXA treatment reversed this effect (Figure 7G,H; Figures S23G,H and S24E,F, Supporting Information).

The in vitro effect of NM@PLGA‐MTI‐OXA on EMT‐related proteins in tumor cells was previously investigated. To further explore the in vivo effects, immunohistochemical staining for N‐cadherin, vimentin, Slug, and Snail proteins was performed on tumor sections from the *Fn*‐infected liver metastasis, AD/*Fn*, and MC38/*Fn* models. In the *Fn*‐infected liver metastasis model, N‐cadherin, Slug, and Snail expression were upregulated in the *Fn* infiltration and OXA treatment groups, whereas NM@PLGA‐MTI‐OXA significantly reversed this upregulation (Figure 7G,I). In the AD/*Fn* model, vimentin, Slug, and Snail expression were upregulated in the *Fn* gavage group; however, treatment groups, excluding the OXA group, exhibited downregulated expression, with NM@PLGA‐MTI‐OXA yielding the most pronounced effect (Figure S23G,I, Supporting Information). Similarly, in the MC38/*Fn* model, *Fn* increased the expression of all four EMT‐related proteins, but this increase was reversed by the different treatments, particularly NM@PLGA‐MTI‐OXA (Figure S24E,G, Supporting Information). Overall, these results indicated that neutrophil membrane‐encapsulated PLGA‐MTI‐OXA effectively reduced EMT‐related protein expression in tumor cells. Given the insignificant effect of OXA on EMT‐related proteins in tumor cells, this therapeutic effect appears contingent upon the clearance of both intra‐ and extracellular bacteria.

## 3 Discussion

Colorectal cancer serves as a prime example of pathogen‐tumor symbiosis, with *Fn* being one of the most well‐established intratumoral pathogens in colorectal cancer, promoting its development.^[^ ^26^ ^]^ In response to this challenge, multiple studies have focused on developing synergistic anti‐tumor strategies targeting *Fusobacterium nucleatum* based on nanomaterials to enhance the efficacy of chemotherapy and immunotherapy. Antibiotic‐based nano delivery systems involve the nanoization of antibiotics and their combination with chemotherapeutic drugs or photothermal agents, synergistically combating bacterial infections and tumors.^[^ ^29^, ^30^ ^]^ For example, Shizhen Geng et al. constructed a system using ferritin‐nanocaged antibiotic and doxorubicin combined with PD‐L1 blocker, which cleared intra‐tumor bacteria and synergistically enhanced immune checkpoint blockade (ICB) therapy.^[^ ^31^ ^]^ Furthermore, the conjugation of *Fn*‐specific bacteriophages with inorganic antimicrobial nanomaterials or chemotherapeutic agents has emerged as a promising approach to selectively target *Fn*‐enriched tumor microenvironments, thereby potentiating chemotherapeutic outcomes via antibacterial synergism.^[^ ^32^, ^33^ ^]^ Additionally, based on the specific binding interactions between surface proteins of *Fn* and tumor cell receptors, Linfu Chen et al. designed and constructed a *Fn*‐mimicking nanomedicine (Colistin‐LipoFM) which selectively killed *Fn* colonizing tumor cells while successfully restoring responsiveness to ICB therapy.^[^ ^27^ ^]^ These pioneering studies have demonstrated the significant advantage of targeting and eliminating intra‐tumor microbiota in treating bacteria‐infiltrated primary tumors. However, to date, few studies have focused on the connection between *Fusobacterium nucleatum* and colorectal cancer liver metastasis, as well as the development of targeted nanomedicines to clear *Fn* and inhibit colorectal cancer liver metastasis. There is an urgent need to base research on tumor biology to develop more precise and effective therapeutic techniques and strategies for CRC liver metastatic therapy.

Our findings utilizing FISH assays on clinical specimens revealed the presence of *Fn* in liver metastatic tissues of advanced colorectal cancer at significantly higher levels compared to adjacent tissues. Furthermore, our results demonstrated a correlation between high *Fn* enrichment in situ and distant metastasis. This observation aligned with previous research by Matthew Meyerson.^[^ ^15^ ^]^ These findings highlighted the potential role of *Fn* in promoting metastatic progression. Our investigation further revealed *Fn* co‐localization with tumor cells, both in clinical specimens and in vitro cell lines. Notably, the primary localization was within the tumor cells themselves. This finding mirrors previous work by Leaf Huang et al., who, through electron microscopy and FISH experiments, observed *Fn* localized in the cytoplasm. Additionally, their research demonstrated that *Fn* could promote liver metastasis in an orthotopic colorectal cancer model.^[^ ^34^ ^]^ Building upon this knowledge, Shang Cai et al. explored the potential role of intracellular bacteria in promoting distant metastasis. Their work demonstrated that intracellular bacteria could assist tumor cells in resisting fluid shear stress by regulating cellular cytoskeletal proteins, thereby aiding in tumor cell survival during metastasis.^[^ ^35^ ^]^ We observed similar changes in the cytoskeleton of tumor cells treated with *Fn*, suggesting a transition from epithelial to mesenchymal phenotypes. Based on these findings, we hypothesize that *Fn* residing within tumor cells might contribute to metastasis, potentially through pathways associated with EMT. Therefore, the clearance of intratumoral bacteria, particularly those residing intracellularly, presents a potentially significant strategy for inhibiting distant metastasis of colorectal cancer.

*Fn*‐induced inflammation may activate and recruit neutrophils to residual tumor tissues. Neutrophil membrane‐based drug delivery, leveraging inflammatory chemotaxis, holds promise for treating *Fn*‐infiltrated colorectal cancer.^[^ ^36^, ^37^ ^]^ Furthermore, our results demonstrated that *Fn* gavage promoted the expression of adhesion molecules, which provided powerful support for Neutrophil membrane‐based drug delivery to effectively target and bind to tumor cells. Therefore, we developed PLGA‐MTI‐OXA nanoparticles coated with neutrophil membrane‐derived vesicles. We observed that chemokine receptors on the neutrophil membrane surface effectively target *Fn*‐induced inflammatory tumor sites by binding to pattern recognition receptors on cell membranes. Subsequently, these nanoparticles are actively internalized by cells, releasing metronidazole to eradicate intracellular bacteria and exert chemotherapeutic effects. This strategy addresses the limitation of metronidazole in effectively eliminating intracellular bacteria within tumor cells, thereby inhibiting the pro‐metastatic role of *Fn*. Aberrant reactivation of EMT is a characteristic feature of tumor metastasis.^[^ ^38^, ^39^ ^]^ We subsequently assessed EMT‐related protein expression and found that *Fn* elimination downregulated their expression, particularly in the NM@PLGA‐MTI‐OXA group, which exhibited the lowest expression levels. This effect may be attributed to the removal of intracellular *Fn*. Furthermore, the observed mechanism of these neutrophil‐mimicking nanoparticles also involves reshaping the immune microenvironment, resulting in a region enriched with CD8^+^ T cells, depleted of MDSCs, and exhibiting reduced release of inflammatory cytokines IL‐6, IL‐10, and IL‐22.

Systemic antibiotic distribution has consistently presented a challenge in clinical practice, particularly due to severe gastrointestinal reactions often associated with intestinal microbiota dysbiosis.^[^ ^40^ ^]^ As illustrated in Figure 6, while the MTI dose did not achieve complete bacteriostasis of *Fn* within the tumor microenvironment, it nonetheless significantly disrupted intestinal microbiota homeostasis. Similarly, noteworthy disruptions in the ecological niche of dominant bacterial species within the fecal microbiota were observed in both the OXA and MTI+OXA groups at standard doses. These observations underscore the adverse effects of the chemotherapeutic agent oxaliplatin on the intestinal microbiota. In contrast, neutrophil‐mimicking nanoparticles effectively cleared intratumoral, and especially intracellular, *Fn* without perturbing the abundance or diversity of the intestinal microbiota.

In conclusion, this study proposes a strategy to disrupt the pathogen‐tumor symbiosis between colorectal cancer and *Fn*, a recognized promoter of tumor development and metastasis. This strategy utilizes FDA‐approved polymeric PLGA nanoparticles encapsulating the antibiotic metronidazole and the chemotherapeutic agent oxaliplatin, facilitating potential clinical translation. Coating these nanoparticles with neutrophil‐derived membrane vesicles enhances targeting and cellular uptake at inflammatory colorectal tumor sites. These neutrophil membrane biomimetic nanoparticles effectively increase the accumulation of metronidazole and oxaliplatin at tumor sites, eliminating both intracellular and extracellular *Fn* within tumor cells. This approach remodels the tumor immune microenvironment, reverses tumor cell EMT, and, importantly, does not disrupt the balance of the intestinal microbiota, thus ensuring drug safety. This study provides a promising cancer treatment strategy focused on targeting the microbiota within the tumor microenvironment, potentially improving chemotherapy efficacy with minimal side effects and demonstrating significant potential for clinical application.

## 4 Experimental Section

### Cell Line and Cell Culture

The CRC cell lines (CT26, MC38, HCT116) were obtained from the State Key Laboratory of Molecular Oncology, National Cancer Center. CT26 and MC38 cells were cultured in Roswell Park Memorial Institute 1640 medium (RPMI 1640; Hyclone), whereas HCT116 cells were cultured in Iscove's Modified Dulbecco's Medium (IMDM; Hyclone). All cells were supplemented with 10% fetal bovine serum (Gibco) and maintained at 37 °C in a humidified atmosphere containing 5% CO_2_.

### Preparation of PLGA‐MTI‐OXA and PLGA‐MTI‐OXA‐Cy5.5

PLGA nanoparticles loaded with MTI and OXA (PLGA‐MTI‐OXA) were prepared using a double emulsion‐solvent evaporation method. PLGA was dissolved in dichloromethane, and a solution of MTI and OXA was added to this solution, followed by ultrasonic emulsification. This primary emulsion was then added dropwise to 5 mL of a 2.5% (w v^−1^) aqueous solution of polyvinyl alcohol (PVA). A secondary emulsion was formed using an ultrasonic probe and subsequently transferred to a 0.25% PVA solution. For PLGA‐MTI‐OXA‐Cy5.5, Cy5.5 was dissolved in dichloromethane, and the remaining steps are the same as above.

### Isolation of Peripheral Neutrophil Membranes (NM)

Neutrophils were isolated from murine bone marrow using a Percoll gradient method, as previously described. Briefly, bone marrow cells were harvested and washed with phosphate‐buffered saline (PBS; Hyclone). The resulting cell pellet was carefully layered onto a two‐layer Percoll gradient consisting of 65% and 55% Percoll diluted in PBS. The gradient was then centrifuged at 2,500 g for 30 min at room temperature. Neutrophils were collected from the interface between the 65% and 55% Percoll layers. Residual erythrocytes were lysed using lysis buffer at 4 °C, yielding highly purified neutrophils. To induce neutrophil activation, cells were incubated with lipopolysaccharide (LPS; 100 ng mL^−1^) for 4 h, followed by a wash with PBS supplemented with a protease inhibitor cocktail. Subsequently, the activated neutrophils were sonicated at 100 W for 60 s (2‐s pulses with 2‐s intervals). The resulting homogenate was then centrifuged at 20 000 g at 4 °C for 30 min to remove unbroken cells and nuclei.

### Synthesis and Characterization of NM@PLGA‐MTI‐OXA

For the synthesis of NM@PLGA‐MTI‐OXA, neutrophil membranes at a concentration of 1 mg mL^−1^ were mixed with PLGA‐MTI‐OXA at 5 mg mL^−1^ at a membrane‐to‐core weight ratio of 1:5. The resulting mixture was then sonicated at 100 W for 6 min. To remove any unbound NM, the product was centrifuged at 14 500 rpm at 4 °C for 50 min.

The size and zeta potentials of the fabricated NM, PLGA‐MTI‐OXA, and NM@PLGA‐MTI‐OXA were determined by DLS using a Zetasizer Nano ZS90 (Malvern Panalytical, UK). Morphology was assessed by uranyl acetate staining and examination via transmission electron microscopy (JEM‐1400 Plus, Japan).

The entrapment efficiency of NM@PLGA‐MTI‐OXA was evaluated. Supernatant and washing solutions from the purification process were collected, and the MTI and OXA content was determined by UV–vis and HPLC, respectively. Entrapment efficiency (%EE) was calculated using the following equation:

(1) $$\%EE=\left(\%\right)\;Amount\;of\;drug\;in\;nanoparticles\;/Amount\;of\;drug\;used\;*100$$

### Cell Proliferation Assay

The in vitro cytotoxicity of NM@PLGA‐MTI‐OXA toward CRC cell lines CT26 and MC38 was assessed using a CCK‐8 cell proliferation assay. Briefly, 2 × 10^4^ cells were seeded in 96‐well plates and cultured overnight. Subsequently, MTI, OXA, PLGA‐MTI‐OXA, and NM@PLGA‐MTI‐OXA were added at the indicated concentrations. Following a 24‐h incubation, 10 µL of CCK‐8 solution was added to each well, and the plates were incubated for an additional 2 h. Absorbance was then measured at 450 nm using a microplate reader.

### Apoptosis Detection

For the apoptosis assay, cells were resuspended in 1× binding buffer and stained with Annexin V‐FITC and propidium iodide (PI) for 10 min at room temperature in the dark. Apoptosis was then assessed by flow cytometry within 1 h.

### Cell Uptake

Cells were seeded in 12‐well plates (Corning, NY, USA) at a density of 2 × 10^5^ cells per well and incubated for 24 h at 37 °C. For cell uptake studies, cells were incubated for 2, 6, and 12 h with free Cy5.5 solution, PLGA‐Cy5.5, and NM@PLGA‐ Cy5.5. At the indicated time points, cells were harvested, washed, fixed, and imaged by the confocal microscope (Leica Microsystems, Germany).

### Immunofluorescence and Confocal Microscopy

Cells were sequentially fixed, permeabilized, and blocked. The primary antibodies applied included anti‐VCAM1 (Ab134047, Abcam), ICAM1 (Ab222736, Abcam), and CD44 (30854‐1‐AP, Proteintech) overnight. Fluorophore‐conjugated secondary antibody (Ab150077, Abcam) was then applied for 1 h. After washing the cells with PBS, they were stained with 4,6‐diamidino‐2‐phenylindole (DAPI) (C1002, Beyotime). The resulting cells were finally captured under the confocal microscope (Leica Microsystems, Germany).

### Wound Healing Assay

A total of 1 × 10^5^ cells were seeded in 6‐well plates and cultured for 12 h. Upon reaching 90% confluence, consistent wounds were created by scratching each well with a 10‐µL pipette tip. Non‐adherent cells were removed by washing twice with PBS. To ensure comparable wound areas, multiple positioning marks were made at the center of the denuded surface. Images of the scratch zones were acquired using inverted microscopy after 12 h.

### Establishment of Animal Models

All animal experiments were conducted in accordance with the guidelines for laboratory animals established by the Chinese Academy of Medical Sciences Cancer Hospital animal laboratory (Beijing, China; ethical approval number: NCC2022A505). To evaluate the effects of the NM@PLGA‐MTI‐OXA combination treatment, a CRC axillary MC38/*Fn* tumor model, a spontaneous AD/*Fn* tumor model, and an *Fn*‐infected liver metastasis model were established. For the CRC axillary MC38/*Fn* tumor model, 1 × 10^6^ MC38 cells (100 µL per mouse) were injected subcutaneously into the right flank of 6‐week‐old male C57BL/6 mice (HFK Bioscience, Beijing, China). *Fn* was injected peritumorally at multiple points every three days, for a total of three injections. Tumor size was measured every three days and calculated using the formula V = 0.5 × L × W^2^, where L and W represent the longest and the shortest tumor lengths.

For the spontaneous AD/*Fn* CRC tumor model, C57BL/6 mice (HFK Bioscience, Beijing, China) received a single intraperitoneal injection of 10 mg kg^−1^ AOM. After a 1‐week recovery period with normal drinking water, mice were administered 2.5% DSS in drinking water for 5 days, followed by 2 weeks of normal drinking water. This cycle was repeated three times. During both DSS administration and the normal drinking water periods, each mouse was orally gavaged three times per week with 1 × 10^7^ CFU of *Fn* to facilitate bacterial infiltration of the colorectal site (AD/*Fn*).

In the *Fn*‐infected liver metastasis model, 1 × 10^6^ CT26‐luc cells, pre‐incubated with *Fn* for 2 h, were injected (100 µL per mouse) subcutaneously into the spleens of 6‐week‐old female BALB/c mice (HFK Bioscience, Beijing, China).

### Biodistribution Analysis in Plasma and Tumor

Biodistribution analyses of MTI+OXA, PLGA‐MTI‐OXA, and NM@PLGA‐MTI‐OXA were conducted using an *Fn*‐infected axillary colorectal cancer model. Prior to the experiment, animals were fasted for 12 h and then randomly divided into three groups receiving a single dose of either MTI+OXA, PLGA‐MTI‐OXA, or NM@PLGA‐MTI‐OXA, corresponding to 25 mg kg^−1^ MTI and 5 mg kg^−1^ OXA. Blood samples were collected via retro‐orbital bleeding and centrifuged at 3000 rpm for 10 min to obtain plasma. Subsequently, mice were euthanized, and tumors were harvested and homogenized with physiological saline at a 1:5 (w v^−1^) ratio. MTI distribution was quantified using Liquid Chromatography‐Mass Spectrometry (LC‐MS), while OXA distribution was quantified using Inductively Coupled Plasma‐Mass Spectrometry (ICP‐MS).

### Flow Cytometry Analysis of Immune Cells In Vivo

Following drug therapy in CRC models, tumor tissues and peripheral blood were freshly harvested. Lymphocytes were isolated from peripheral blood via red blood cell lysis. Subsequently, cells were collected and stained with specific fluorophore‐conjugated antibodies for flow cytometric analysis using a BD FACS Aria™ III. Briefly, surface antibody staining was performed for 30 min at 4 °C in the dark. The following cell populations were identified based on cell marker expression: CD4^+^ T cells (CD45^+^CD3^+^CD4^+^), CD8^+^ T cells (CD45^+^CD3^+^CD8^+^), and MDSCs (CD45^+^ CD11b^+^ Gr‐1^+^). Data were analyzed using FlowJo v.10.4 software.

### Immunohistochemistry Assay

Following deparaffinization in xylene and rehydration through a graded alcohol series, antigen retrieval was performed by heating in citrate buffer (pH 6.0). Endogenous peroxidase activity was quenched by incubation with 0.3% hydrogen peroxide for 10 min, and non‐specific binding was blocked with 10% normal animal serum for 10 min. Subsequently, slides were incubated with primary antibodies at 4 °C for 24 h. After incubation with biotinylated secondary antibodies and horseradish peroxidase‐conjugated avidin, staining was developed using 3,3'‐Diaminobenzidine (DAB) and counterstained with hematoxylin. Finally, slides were dehydrated, coverslipped, and imaged using a pathological workstation.

### 16S rDNA Sequencing Assay

Fecal samples from MC38/*Fn* tumor model and AD/*Fn* CRC spontaneous model mice were collected following administration of various treatments: PBS, *Fn* (10^7^ CFU per mouse), MTI (25 mg kg^−1^), OXA (5 mg kg^−1^), MTI + OXA combination, PLGA‐MTI‐OXA, and NM@PLGA‐MTI‐OXA. Subsequently, these samples were subjected to 16S ribosomal RNA (16S rRNA) gene sequencing. Data analysis was performed on the Majorbio Cloud Platform, a freely accessible online platform (https://cloud.majorbio.com/).

### Fluorescent In Situ Hybridization (FISH)

This research was approved by the Ethics Committee of Cancer Hospital (Beijing, China; approval number: 2021071216433802). Clinicopathological data (age, sex, stage, and TNM stage) were available for all specimens. A FAM‐conjugated *Fn* probe (5′‐CTTGTAGTTCCGCYTACCTC‐3′) was labeled with Spectrum‐Green to detect *Fn*. A Cy5‐conjugated EUB338 universal bacterial probe (5′‐GCTGCCTCCCGTAGGAGT‐3′) was labeled with Spectrum‐Red as a positive control. FISH was performed according to a previously published protocol. Subsequently, a PerkinElmer Vectra Polaris system was used for panoramic scans, and CLSM was used to image randomly selected fields. Finally, ImageJ software (Fiji 1.46, National Institutes of Health) was used to quantify *Fn* enrichment.

### Immunostaining Analysis

Slides were independently evaluated by two pathologists. Protein expression levels were quantified using a staining index (SI) calculated as the product of staining intensity and the percentage of positive cells. Staining intensity was graded on a four‐tier scale: 0 (no staining), 1 (weak staining), 2 (moderate staining), and 3 (strong staining). The percentage of positive cells was scored as: 0 (0%), 1 (1–30%), 2 (31–50%), and 3 (>50%). Therefore, SI = (intensity score) × (positive staining score).

### Data Analysis

Statistical analyses were performed using SPSS version 23.0 (IBM Corp., Armonk, NY, USA) and GraphPad Prism version 8.0 (GraphPad Software, LLC, San Diego, CA, USA). All experiments were conducted in triplicate. Data in bar graphs are presented as mean ± standard deviation (SD). Comparisons between groups were performed using Student's t‐test, one‐way ANOVA, or the χ^2^ test, as appropriate. A *p*‐value<0.05 was considered statistically significant.

## Conflict of Interest

The authors declare no conflict of interest.

## Supporting information



Supporting Information

## Data Availability

The data that support the findings of this study are available from the corresponding author upon reasonable request.
