# Supplementary material for: Neutrophil‐Mimicking Nanomedicine Eliminates Tumor Intracellular Bacteria and Enhances Chemotherapy on Liver Metastasis of Colorectal Cancer
Source: Adv Sci (Weinh). 2025 May 28;12(30):e04188. doi: 10.1002/advs.202504188 (PMC12376532; doi:10.1002/advs.202504188)
Supplement: Supplementary file 1 — Supporting Information [file ADVS-12-e04188-s001.docx]

**Supplementary information**

**Neutrophil-Mimicking Nanomedicine Eliminates Tumor Intracellular Bacteria and Enhances Chemotherapy on Liver metastasis of Colorectal cancer.**

Yanan Niu^1#^, Xu Zhao^1#^, Yong Li^2^, Xiaoya Ma^1^, Weifeng Yang^3^, Jie Ma^4^*, Wanglin Li^3^*, Wei Yuan^1^*

1. State Key Laboratory of Molecular Oncology, National Cancer Center/Cancer Hospital, Chinese Academy of Medical Sciences, Peking Union Medical College, Beijing, 100021, P.R. China.
2. Department of Thoracic Surgery, National Cancer Center/Cancer Hospital, Chinese Academy of Medical Sciences, Peking Union Medical College, Beijing, 100021, P.R. China.
3. Department of Gastrointestinal Surgery, Huadu District Peoples' Hospital of Guangzhou, 48 Xinhua Road, Huadu District, Guangzhou, 510800, P.R. China.
4. Department of Biotherapy, Beijing Hospital, National Center of Gerontology, Institute of Geriatric Medicine, Chinese Academy of Medical Sciences, Graduate School of Peking Union Medical College, Beijing, 100730, P.R. China.

*Corresponding author:

Wei Yuan：yuanwei@cicams.ac.cn

Jie Ma：majie4685@bjhmoh.cn

Wanglin Li：eylwl@scut.edu.cn

^#^These authors contributed equally to this work.

**Content**

**Figure S1** Representative images of CT26 cell treated with *Fn*.

**Figure S2** Western blot identification of membrane-associated adhesive proteins including VCAM1, CD44, ICAM1 in intestinal tract tissue.

**Figure S3** Characterization of neutrophil-mimicking nanoparticles.

**Figure S4** Neutrophil-mimicking nanoparticles stability study *in vitro*.

**Figure S5** Quantitative analysis of fluorescence intensity in Figure2E.

**Figure S6** The confocal images of the colocalization of NM@PLGA-Cy5.5 (red) and the immunofluorescence stain of VCAM1, CD44 and ICAM1 (green) on CT26 cells treated with or without *Fn*.

**Figure S7** The confocal images of the nanoparticles(red) and the immunofluorescence stain of VCAM1, CD44 and ICAM1 (green) on CT26 cells treated with *Fn*.

**Figure S8** The confocal images of the nanoparticles (red) and lysosome (Lysotracker dye-green).

**Figure S9** Fluorescence intensity in vital organs and colon treated with different group at 6h.

**Figure S10** Quantitative analysis of fluorescence intensity in Figure 2F.

**Figure S11** Distribution of MTI and OXA in plasma and tumor after 6h post-injection of MTI+OXA, PLGA-MTI-OXA and NM@PLGA-MTI-OXA.

**Figure S12** Quantitative analysis of diameter of inhibition zone in figure3A.

**Figure S13** The intracellular behavior of neutrophil-mimicking nanoparticles *in vitro*.

**Figure S14** Quantitative analysis of *Fn* colony numbers in figure3H.

**Figure S15** The gray value ratio of EMT related proteins in CT26 and MC38 in Figure 3I.

**Figure** **S16** Representative images of CT26 and HCT116 cell treated with *Fn*.

**Figure S17** Body weight fluctuation.

**Figure S18** Biosafety evaluation of AD/*Fn* CRC spontaneous model.

**Figure S19** Neutrophil-Mimicking nanoparticles can effectively delay the progression of MC38/*Fn* axillary colorectal cancer model.

**Figure S20** Biosafety evaluation of MC38/*Fn* axillary colorectal cancer model.

**Figure S21** IVIS images of bioluminescence signal of luciferase assay and quantitative analysis for each group.

**Figure S22** Neutrophil-mimicking nanoparticles maintained intestinal flora balance in MC38/*Fn* axillary colorectal cancer model.

**Figure S23** Neutrophil-mimicking nanoparticles remodeled the tumor immune microenvironment and reversed the *Fn*-mediated EMT process in AD/*Fn* CRC spontaneous model.

**Figure S24** Neutrophil-mimicking nanoparticles remodeled the tumor immune microenvironment and reversed the *Fn*-mediated EMT process in MC38/*Fn* axillary colorectal cancer model.

**Figure S25** Flow cytometry in peripheral blood of different models.

**Table S1** Correlation between *Fn* enrichment level and clinicopathological characteristics in CRC cases.

**Figure S1**


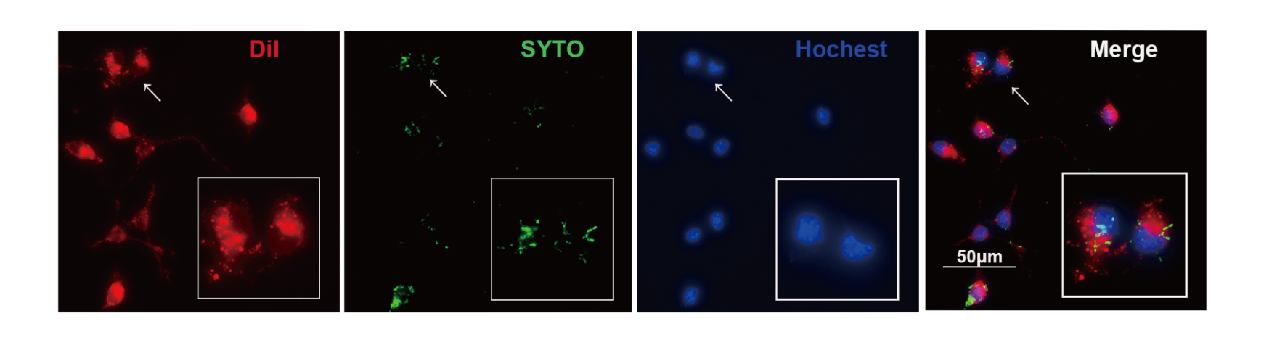


**Figure S1** Representative images of CT26 cell treated with *Fn.* Cytoskeleton was stained with DiI (red), *Fn* was stained with SYTO (green) and cell nucleoid was stained with hochest (blue). Scale bar = 50 μm.

**Figure S2**


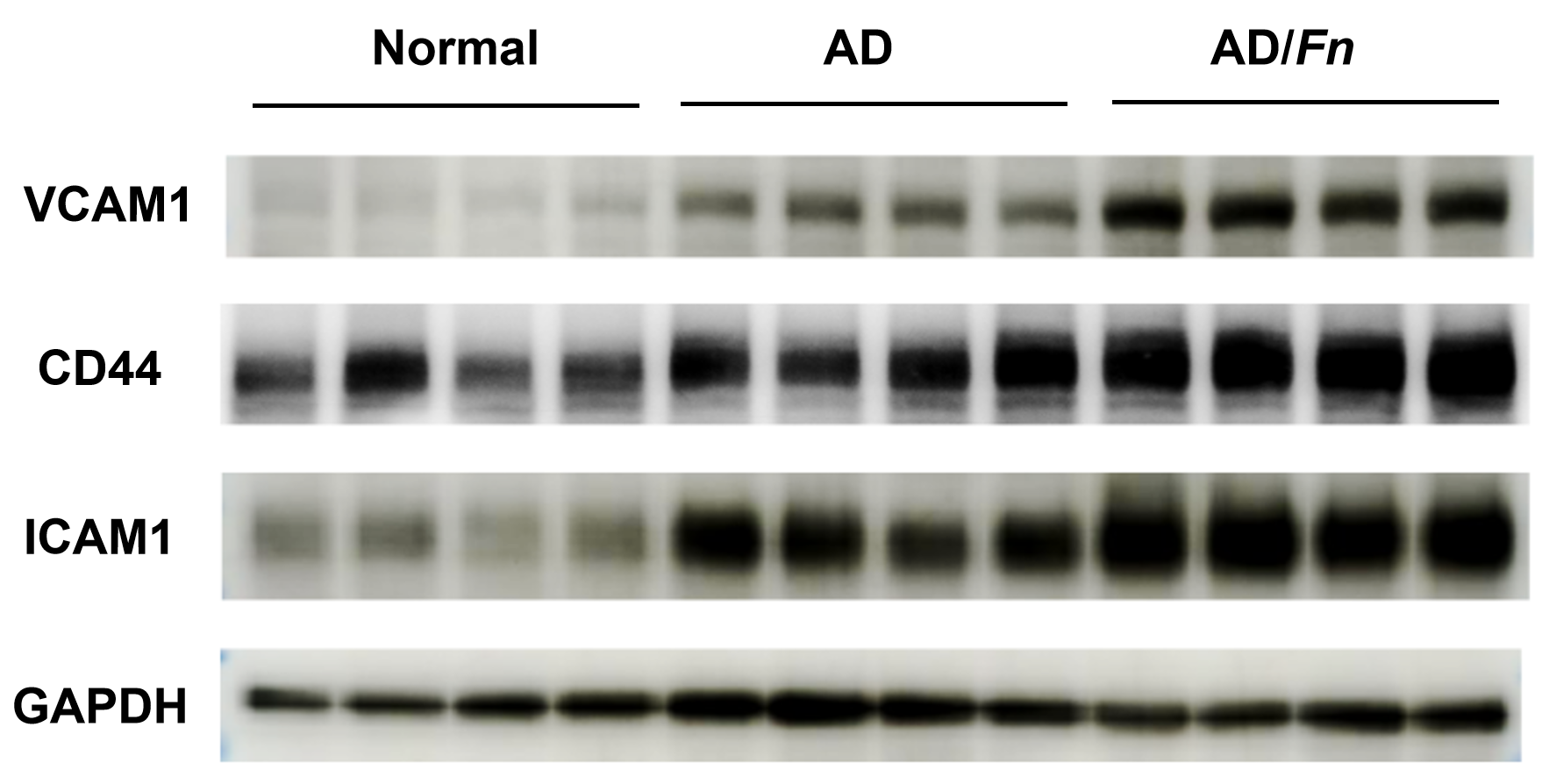


**Figure S2** Western blot identification of adhesion molecules including VCAM1, CD44, ICAM1 in intestinal tract tissue.

**Figure S3**


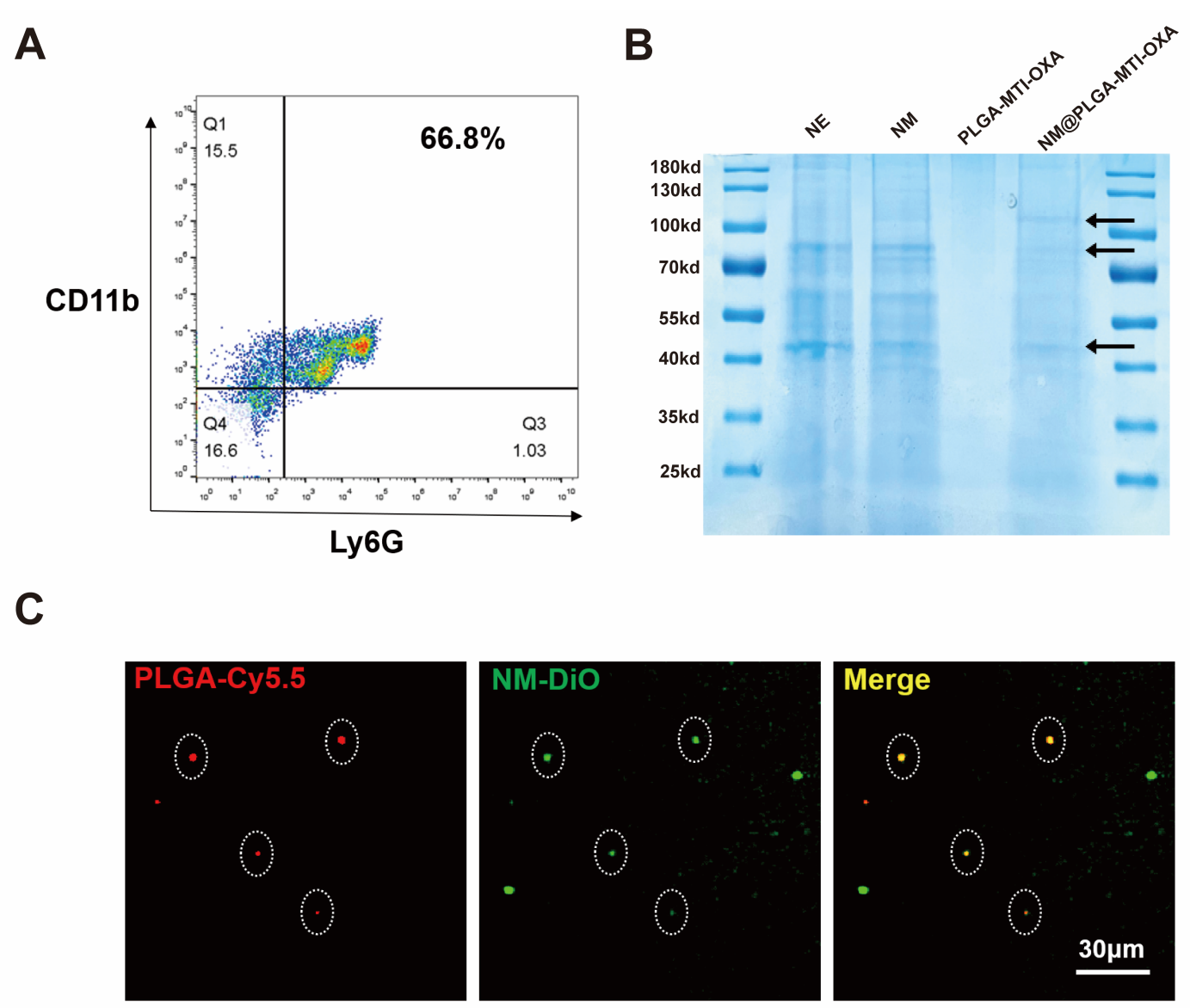


**Figure S3** Characterization of neutrophil-mimicking nanoparticles.

**(A)** Flow cytometry of Neutrophil from mouse bone marrow. **(B)** Coomassie brilliant blue staining for total proteins of Neutrophil, Neutrophil membrane (NM), PLGA-MTI-OXA and NM@ PLGA-MTI-OXA. **(C)** Representative images of NM@PLGA-Cy5.5. Neutrophil membrane was stained with DiO (green). Scale bar = 30 μm.

**Figure S4**


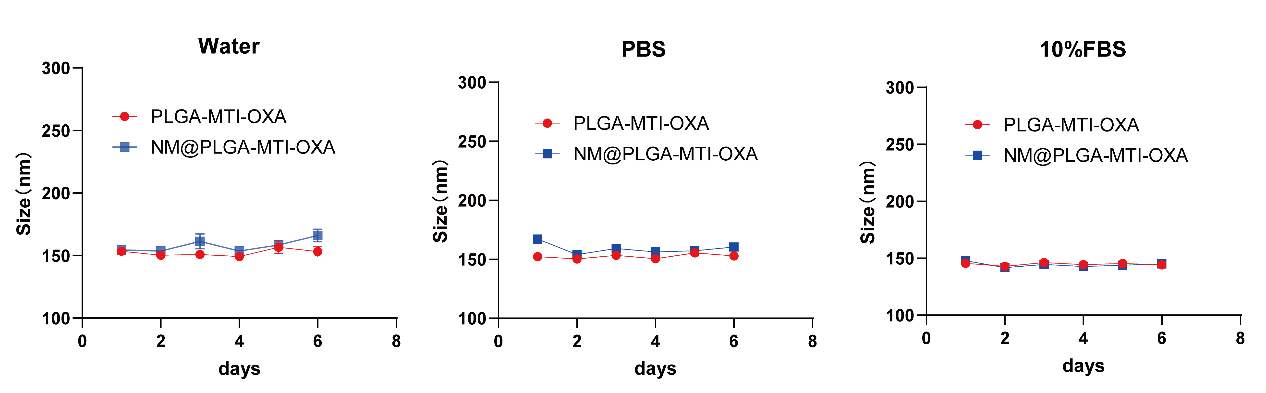


**Figure S4** Neutrophil-mimicking nanoparticles stability study *in vitro*. Stability of PLGA-MTI-OXA and NM@PLGA-MTI-OXA over time in water, PBS and 10%FBS (n = 3).

**Figure S5**


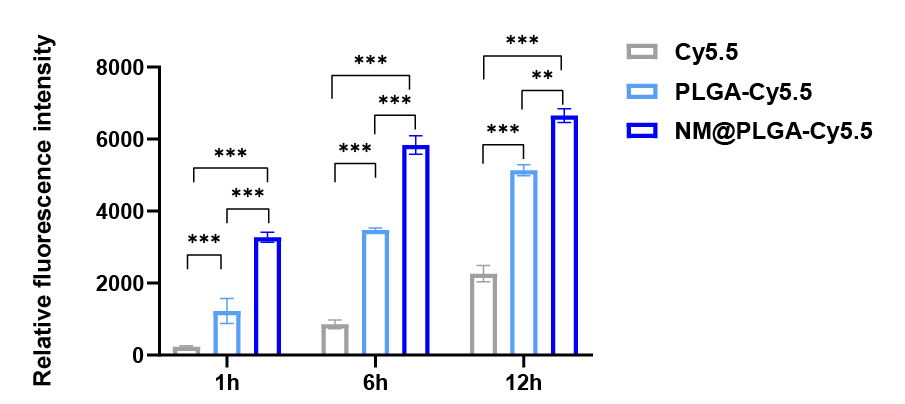


**Figure S5** Quantitative analysis of fluorescence intensity in Figure2E (n=3). * *P* < 0.05; ** *P* < 0.01; *** *P* < 0.001.

**Figure S6**


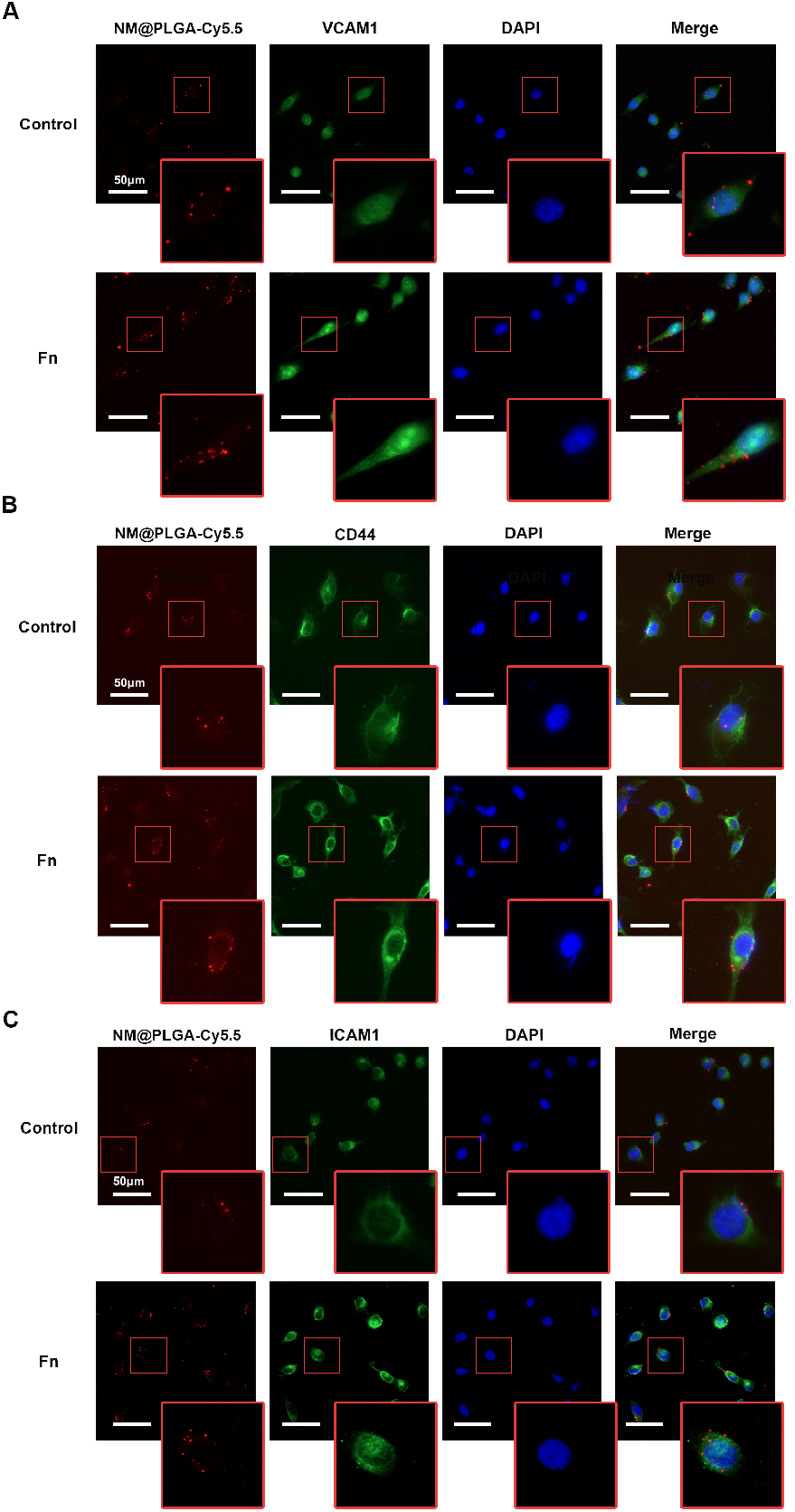


**Figure S6** The confocal images of the colocalization of NM@PLGA-Cy5.5 (red) and the immunofluorescence stain of VCAM1, CD44 and ICAM1 (green) on CT26 cells treated with or without *Fn*. blue: DAPI-labeled nuclei. Scale bar= 50 μm.

**Figure S7**

**
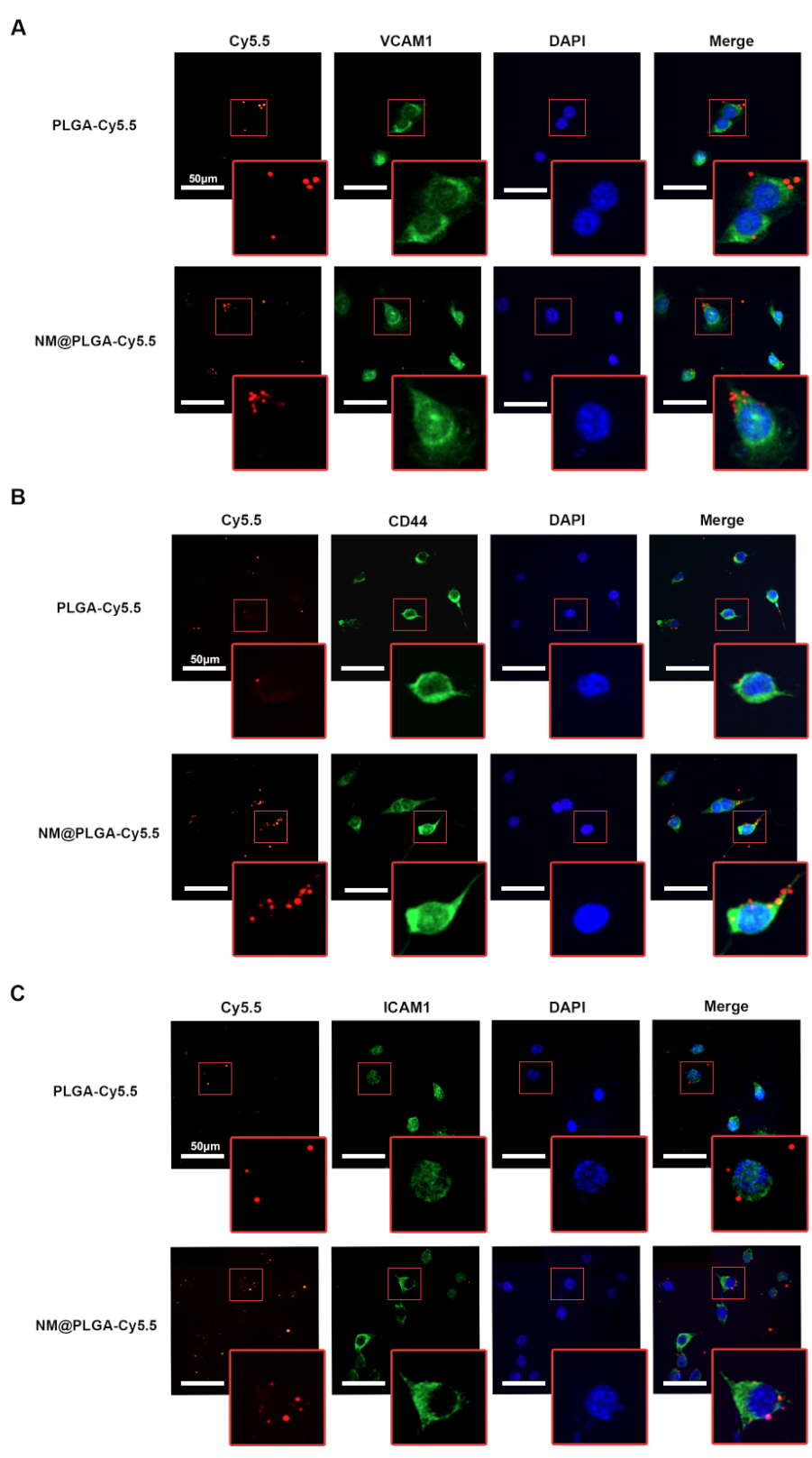
**

**Figure S7** The confocal images of the nanoparticles(red) and the immunofluorescence stain of VCAM1, CD44 and ICAM1 (green) on CT26 cells treated with *Fn*. blue: DAPI-labeled nuclei. Scale bar= 50 μm.

**Figure S8**


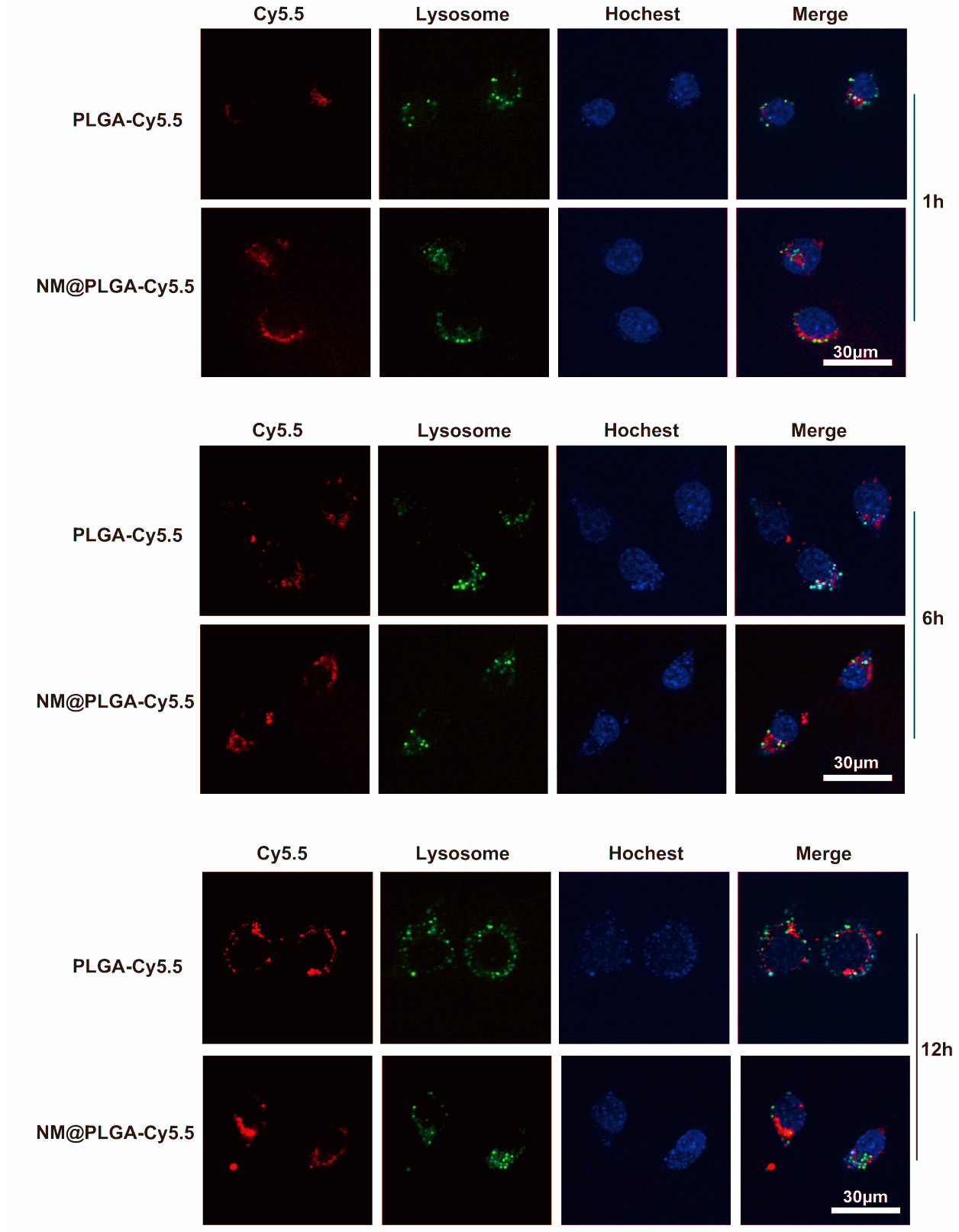


**Figure S8** The confocal images of the nanoparticles (red) and lysosome (Lysotracker dye-green). blue: Hochest-labeled nuclei. Scale bar= 30 μm.

**Figure S9**


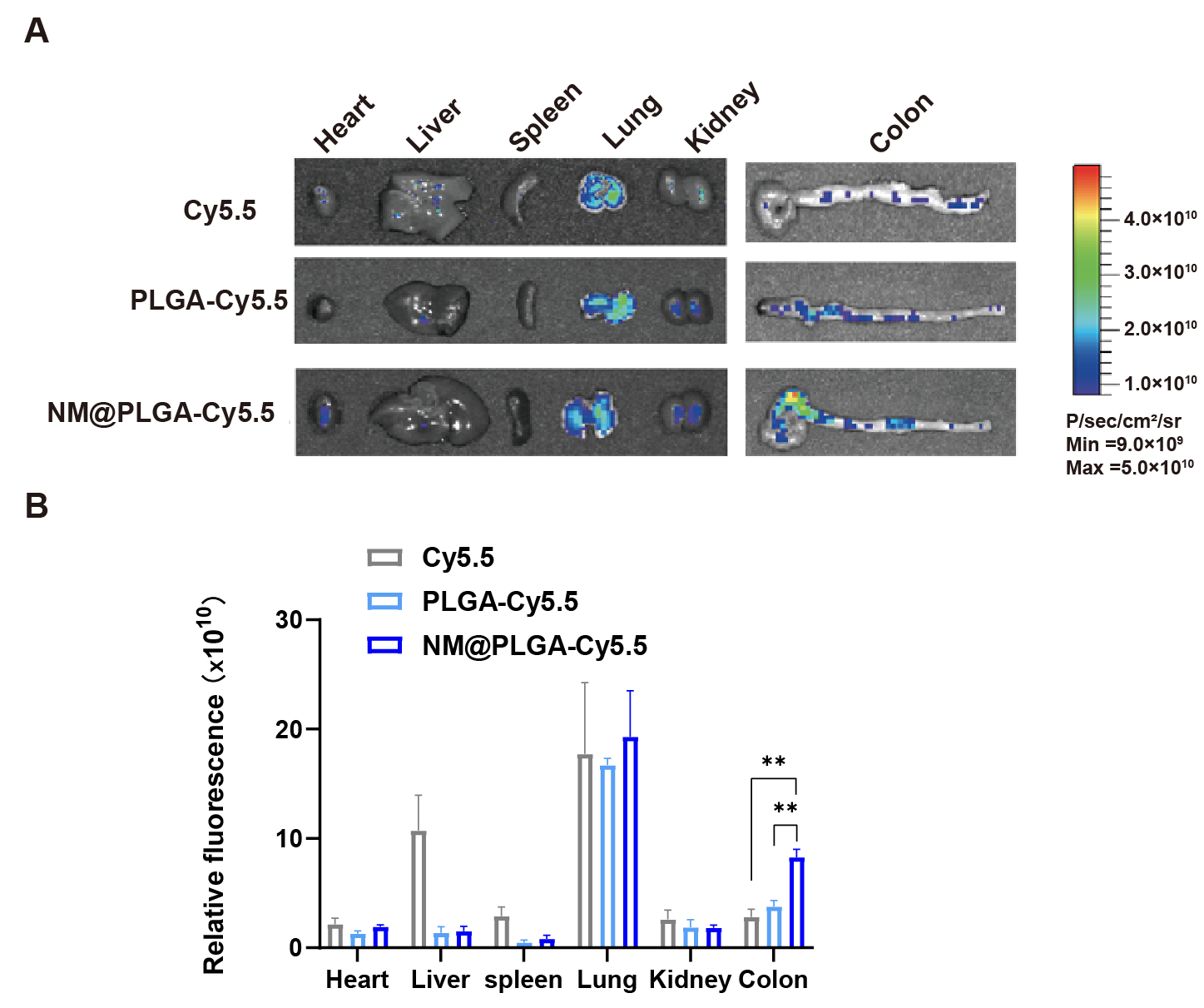


**Figure S9** Fluorescence intensity in vital organs and colon treated with different group at 6h. **(A)**Representative images of vital organs and colon after 6h post-injection of Cy5.5, PLGA-Cy5.5 and NM@PLGA-Cy5.5. **(B)** Quantitative analysis of fluorescence intensity in (A) (n=3). * *P* < 0.05; ** *P* < 0.01; *** *P* < 0.001.

**Figure S10**


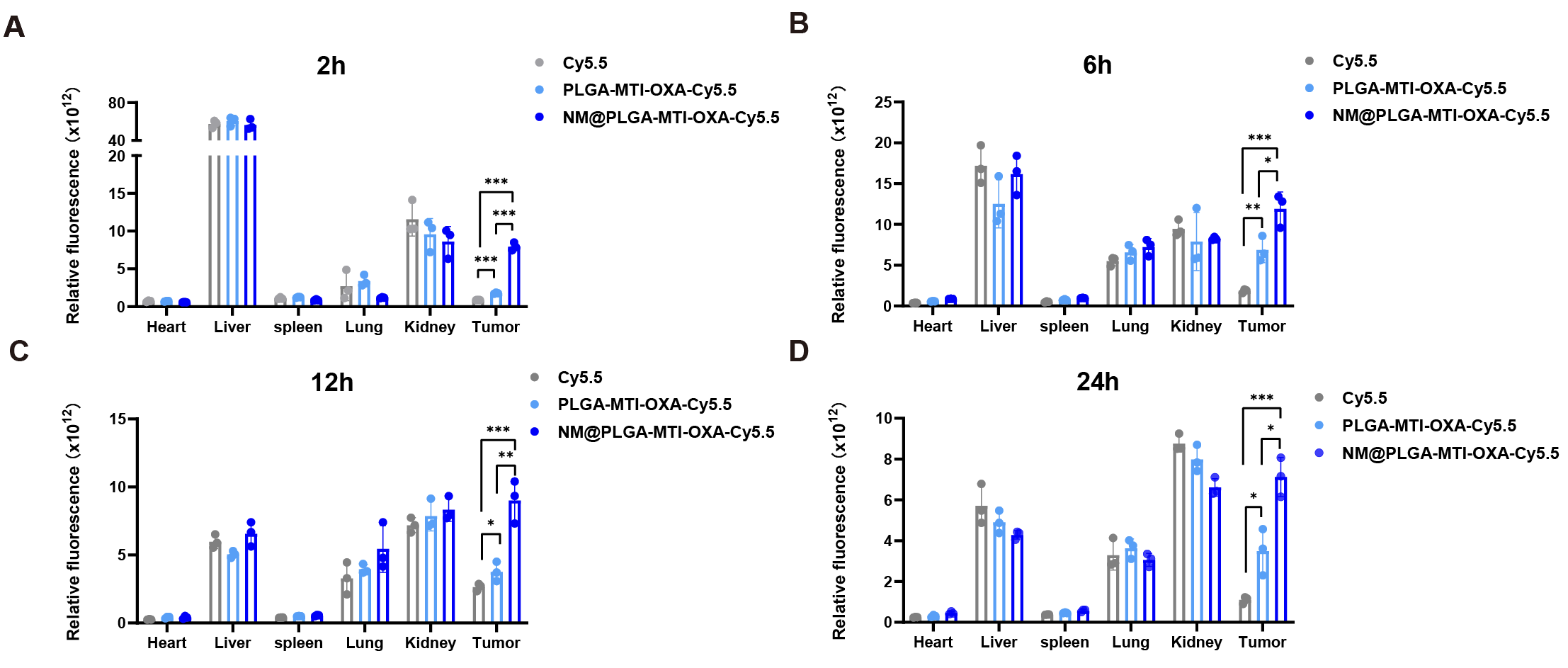


**Figure S10** Quantitative analysis of fluorescence intensity in Figure 2F (n=3). * *P* < 0.05; ** *P* < 0.01; *** *P* < 0.001.

**Figure S11**


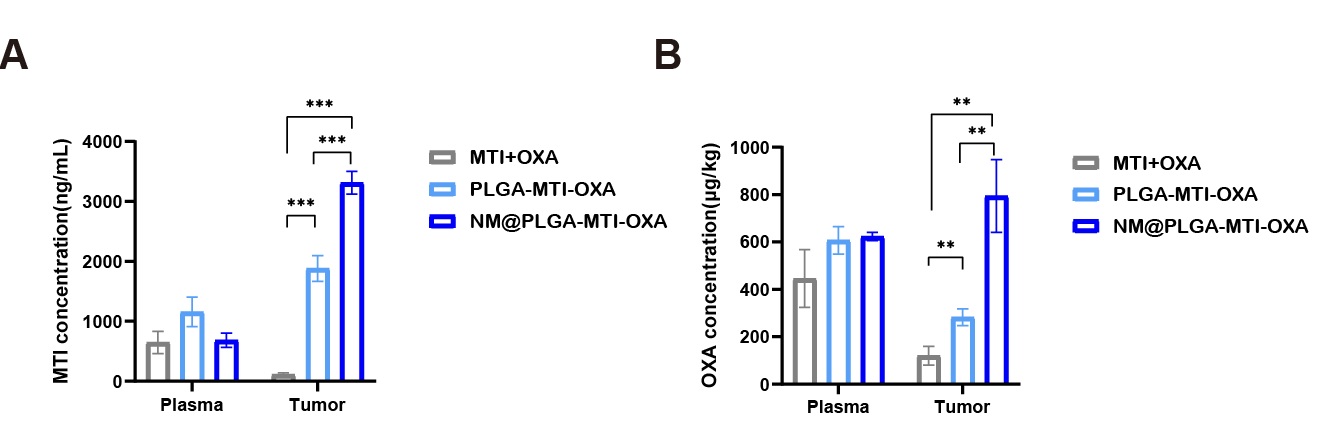


Figure S11 Distribution of MTI**(A)** and OXA**(B)**in plasma and tumor after 6h post-injection of MTI+OXA, PLGA-MTI-OXA and NM@PLGA-MTI-OXA (n=3). * *P* < 0.05; ** *P* < 0.01; *** *P* < 0.001.

**Figure S12**


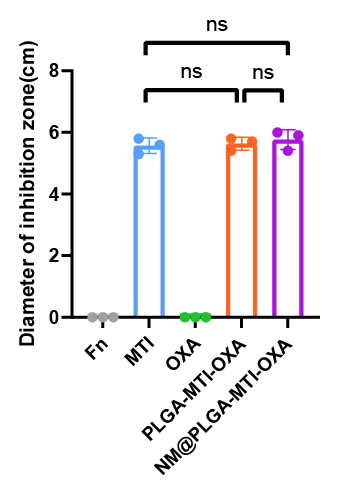


Figure S12 Quantitative analysis of diameter of inhibition zone in figure3A (n = 3).

**Figure S13**


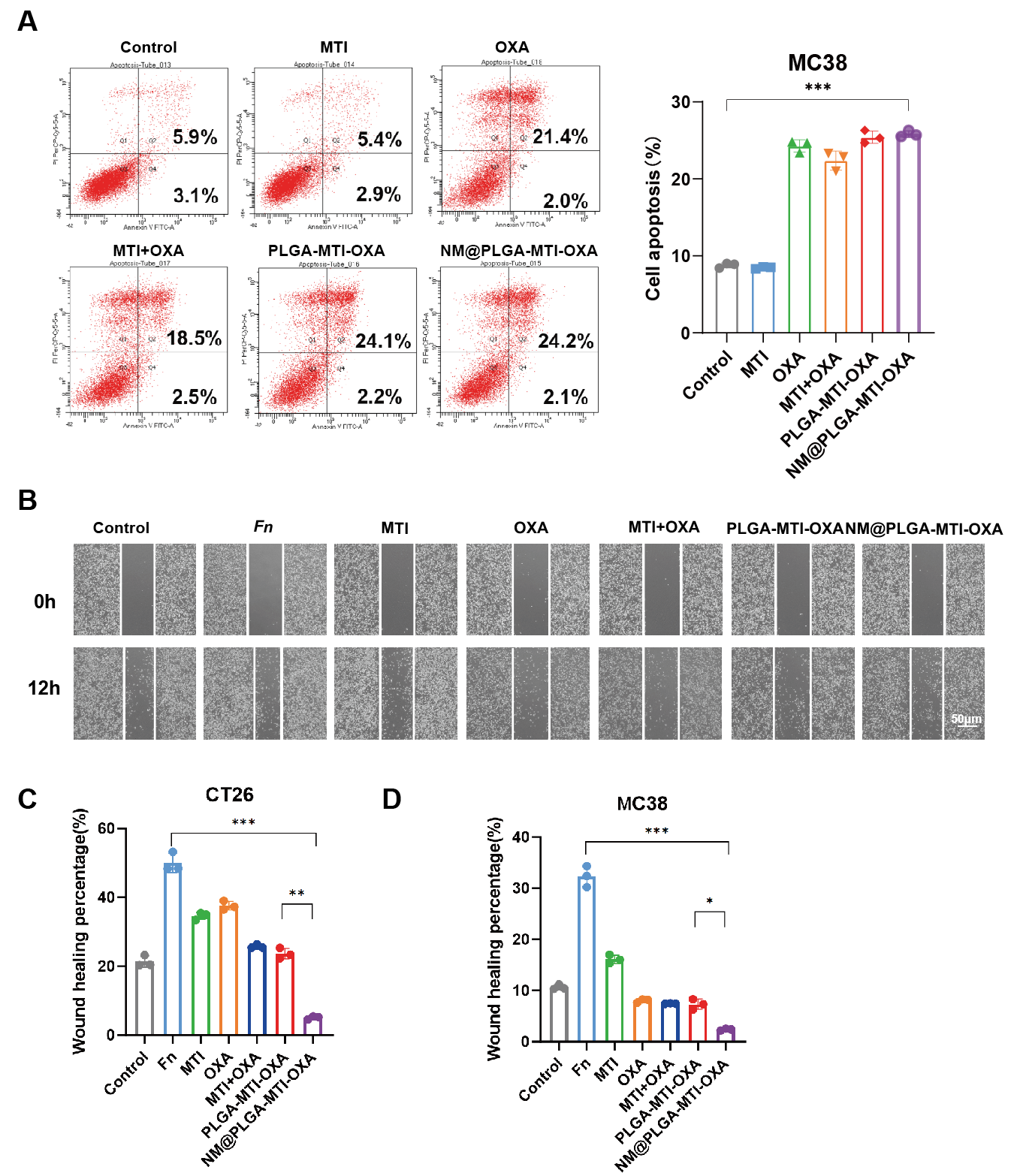


**Figure S13** The intracellular behavior of neutrophil-mimicking nanoparticles *in vitro*.

**(A)** Flow cytometry analysis with Annexin V/PI staining evaluating the percentages of apoptotic cells of MC38 cells among different drug treatment groups. **(B)** Wound healing assay of MC38 cells treated with different drugs for 12 h. Scale bar= 50 μm. **(C)**Quantitative analysis of wound healing percentage in Figure3G (n=3). **(D)**Quantitative analysis of wound healing percentage in (B) (n=3). * *P* < 0.05; ** *P* < 0.01; *** *P* < 0.001.

**Figure S14**


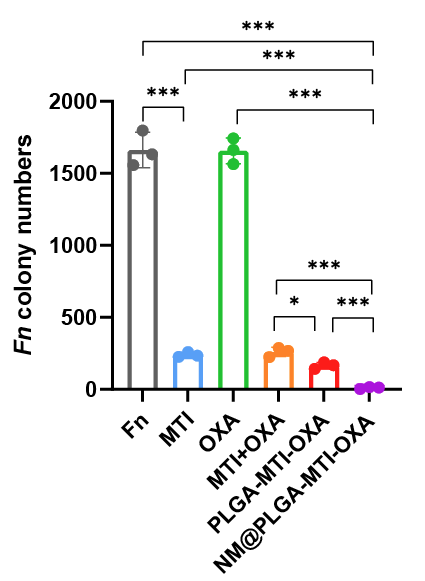


**Figure S14** Quantitative analysis of *Fn* colony numbers in figure3H (n = 3). * *P* < 0.05; ** *P* < 0.01; *** *P* < 0.001.

**Figure S15**


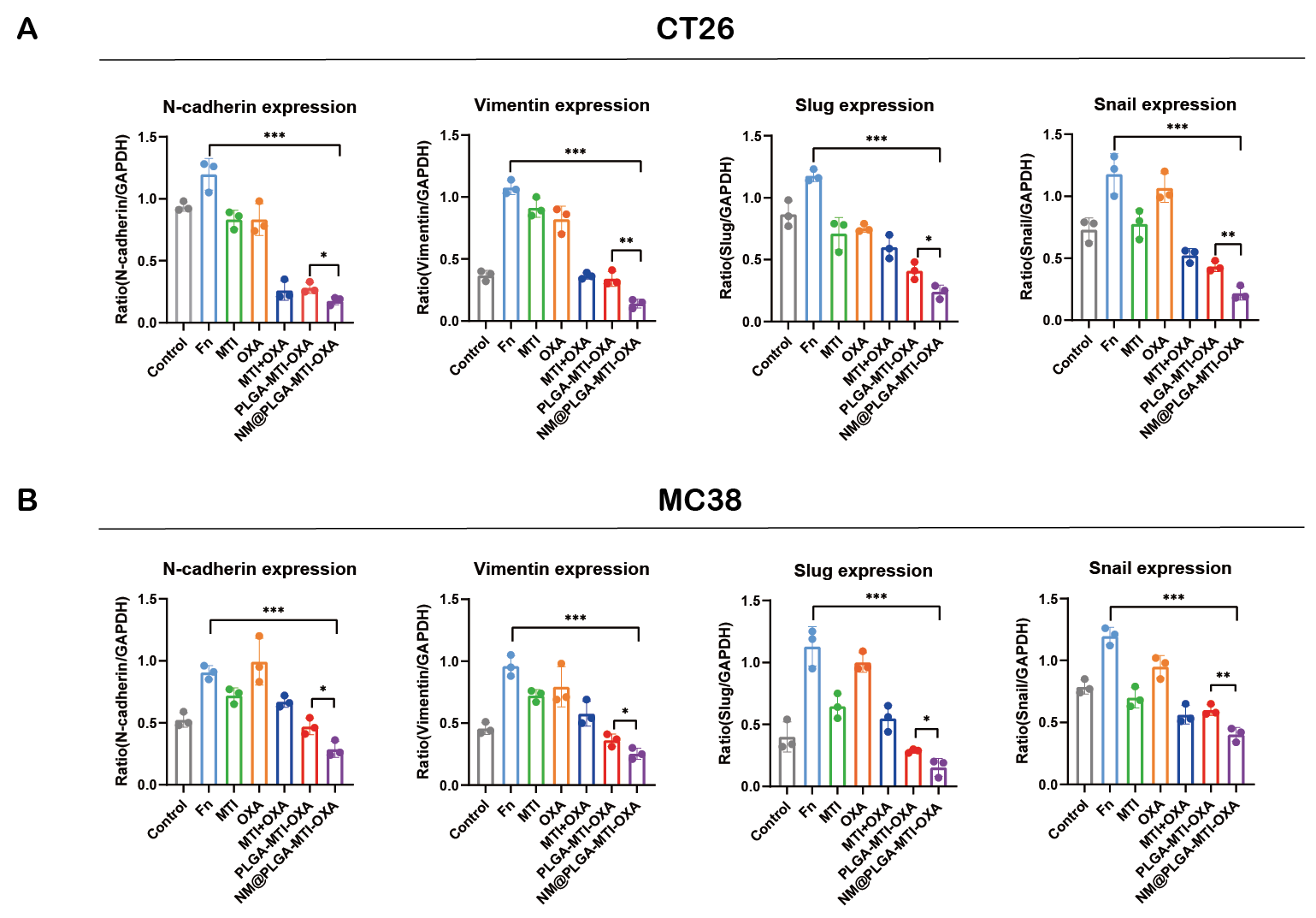


**Figure S15** The gray value ratio of EMT related proteins in CT26 **(A)** and MC38 **(B)** in Figure 3I (n = 3). * *P* < 0.05; ** *P* < 0.01; *** *P* < 0.001.

**Figure S16**


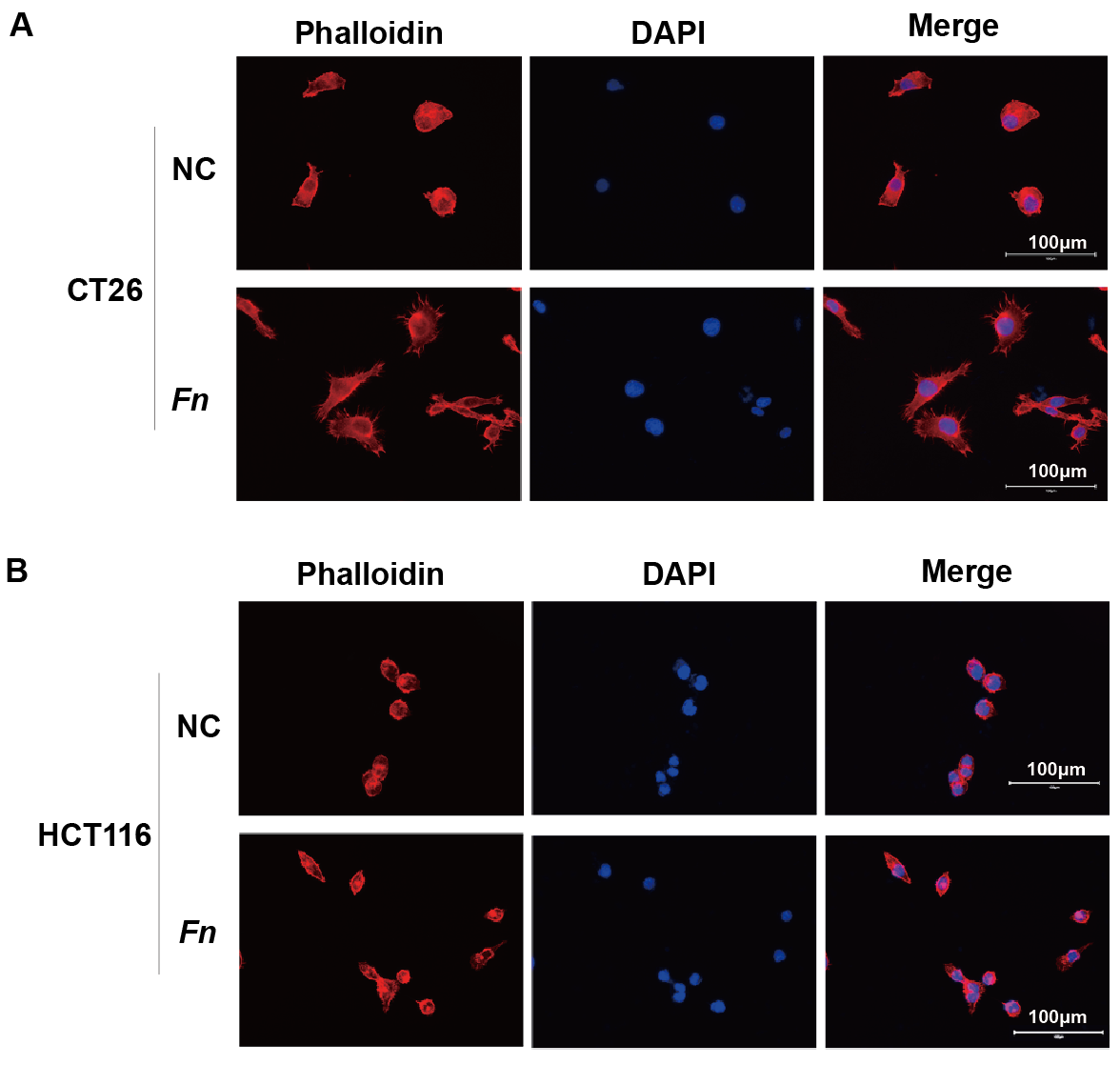


**Figure S16** Representative images of CT26(A) and HCT116 cell(B) treated with *Fn*. Cytoskeleton was stained with phalloidin (red) and cell nucleoid was stained with DAPI (blue). Scale bar = 100 μm. * *P* < 0.05; ** *P* < 0.01; *** *P* < 0.001

.

**Figure S17**


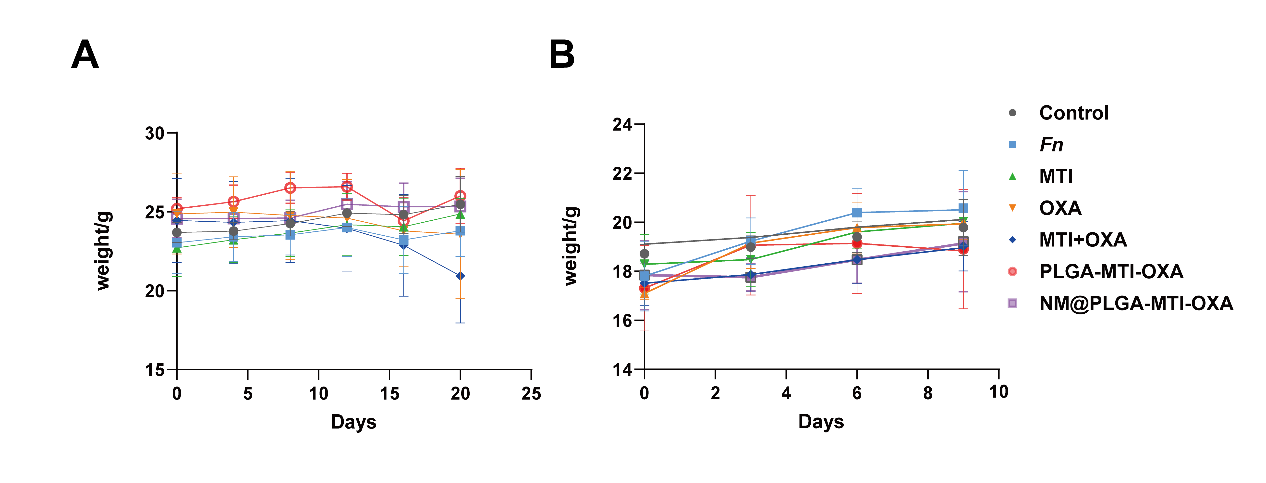


**Figure S17** Body weight fluctuation. (A) Body weight curves of AD/*Fn* CRC spontaneous model with various treatments(n = 5). (B) Body weight curves of *Fn*-infected liver metastasis model with various treatments(n = 3).

**Figure S18**


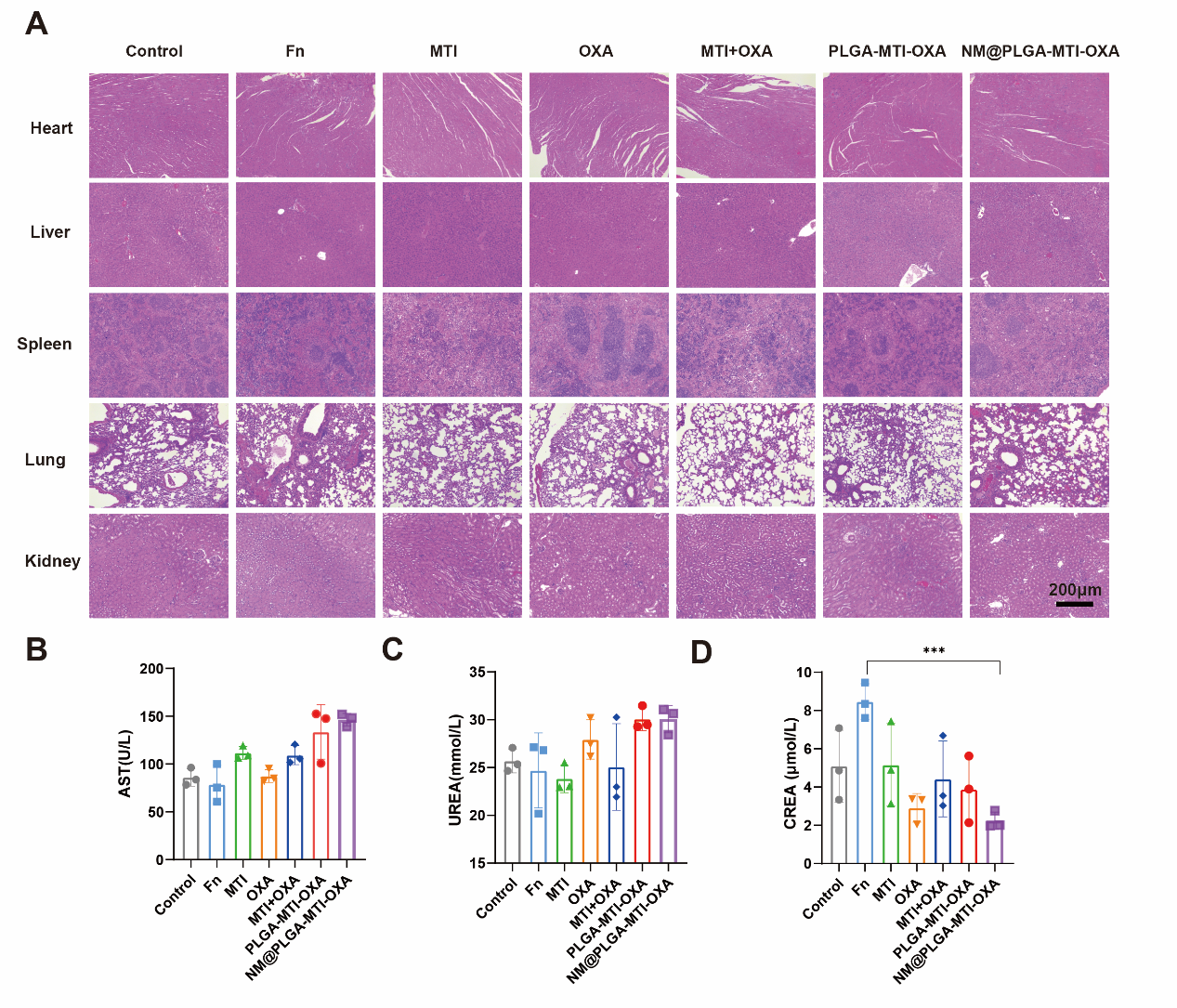


**Figure S18** Biosafety evaluation of AD/*Fn* CRC spontaneous model.

**(A)**H&E staining images of major organs of mice after different treatments, scale bar: 100 μm. The biochemistry level of liver and renal function after different treatments: **(B)** aspartate transaminase (AST), **(C)** urease (UREA), **(D)** creatinine (CREA) (n = 3). * *P* < 0.05; ** *P* < 0.01; *** *P* < 0.001.

**Figure S19**


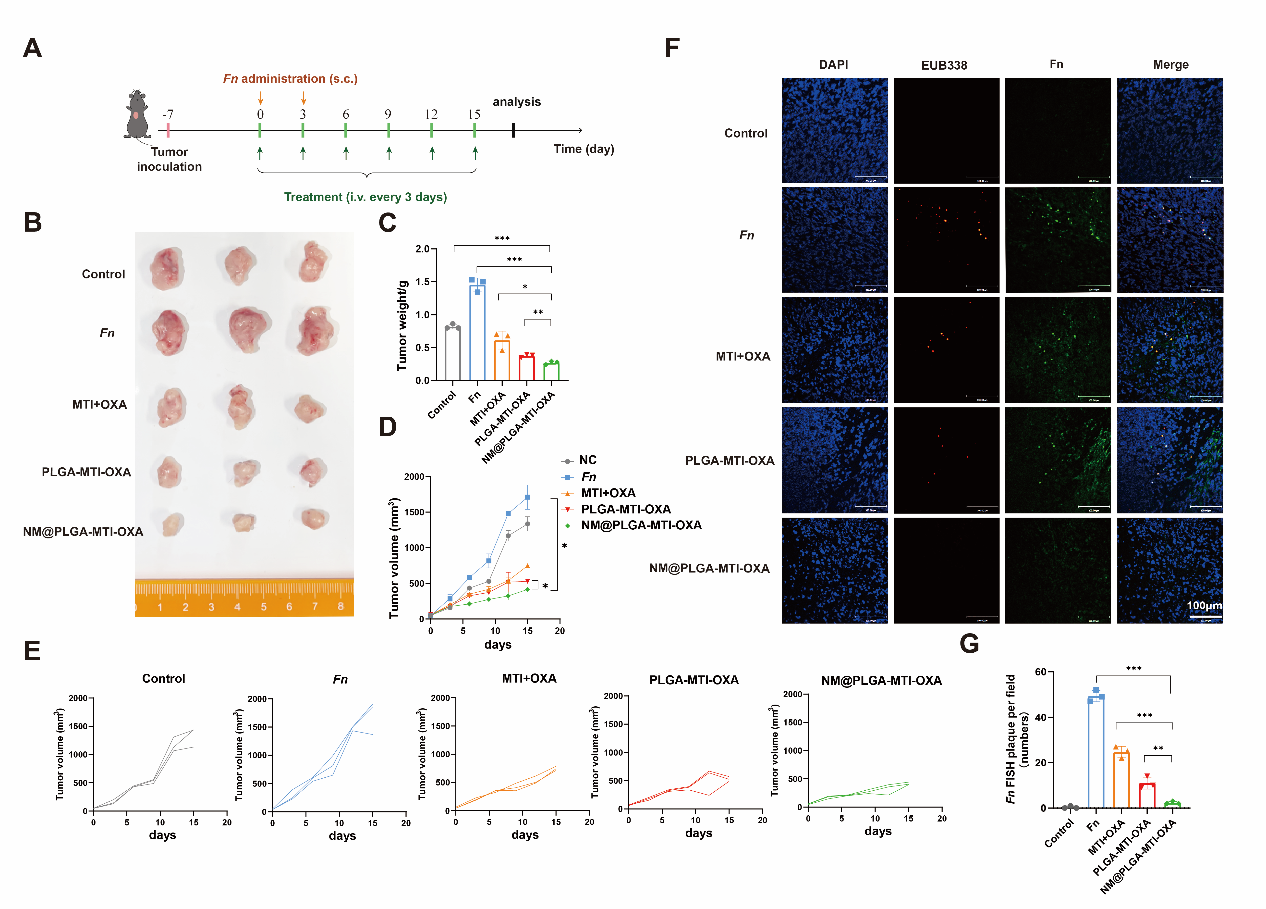


**Figure S19** Neutrophil-Mimicking nanoparticles can effectively delay the progression of MC38/*Fn* axillary colorectal cancer model.

**(A)** Timeline of the MC38/*Fn* axillary colorectal cancer model in vivo treatment. **(B)** The representative tumor images of different treatment group. **(C)** Tumor weight of different treatment group. **(D)** Change curve of tumor growth in different treatment group. **(E)** Change curve of individual tumor growth. **(F)** Photographs of CLSM taken by FISH to detect *Fn* infiltration in sites of tumors, blue: DAPI-labeled nuclei, red: CY5-EUB338 universal bacterial probes, green: FAM-FUS664 probe, scale bar =100 μm (n = 3). (G) Quantitative analysis of *Fn* FISH plaques in F. * *P* < 0.05; ** *P* < 0.01; *** *P* < 0.001.

**Figure S20**


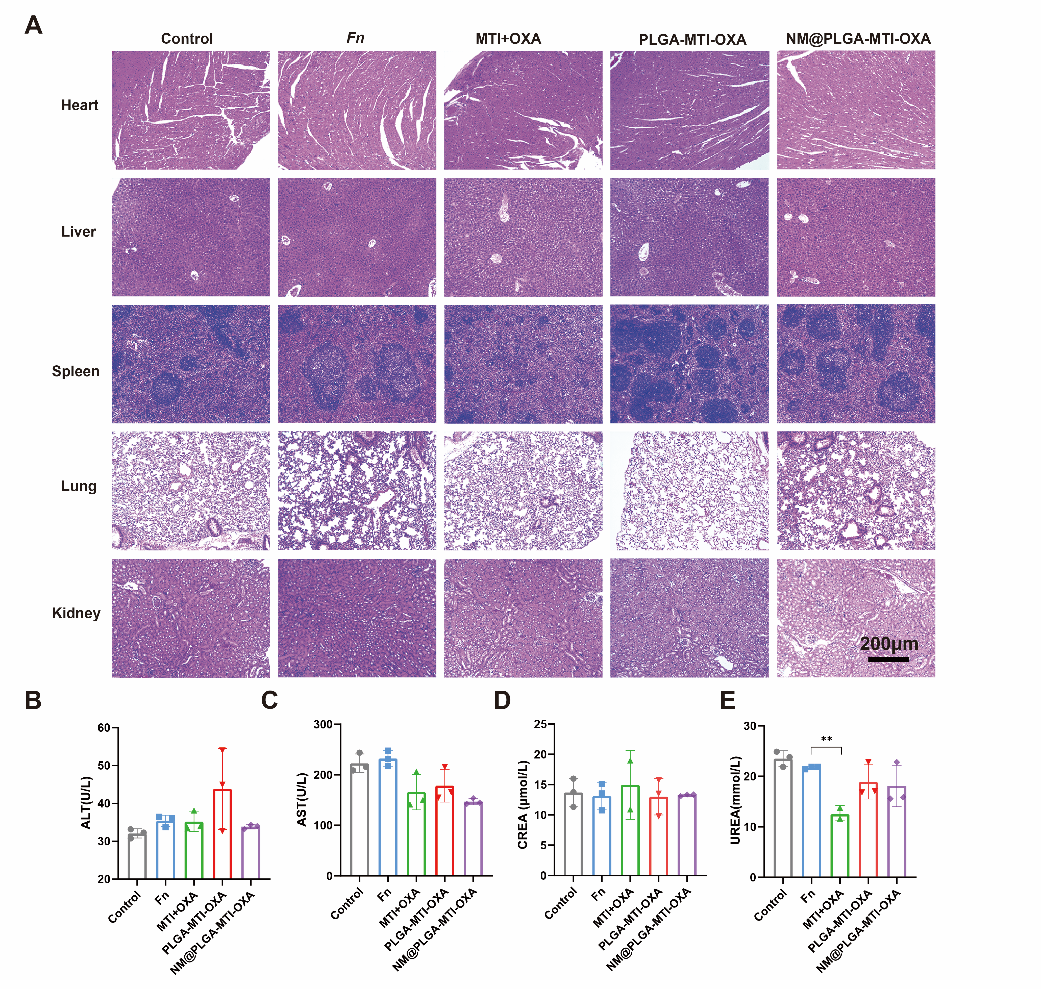


**Figure S20** Biosafety evaluation of MC38/*Fn* axillary colorectal cancer model.

**(A)**H&E staining images of major organs of mice after different treatments. Scale bar: 100 μm. The biochemistry level of liver and renal function after different treatments:**(B**

**)** alanine transaminase (ALT), **(C)** aspartate transaminase (AST), **(D)** creatinine (CREA), **(E)** urease (UREA) (n = 3). * *P* < 0.05; ** *P* < 0.01; *** *P* < 0.001.

**Figure S21**


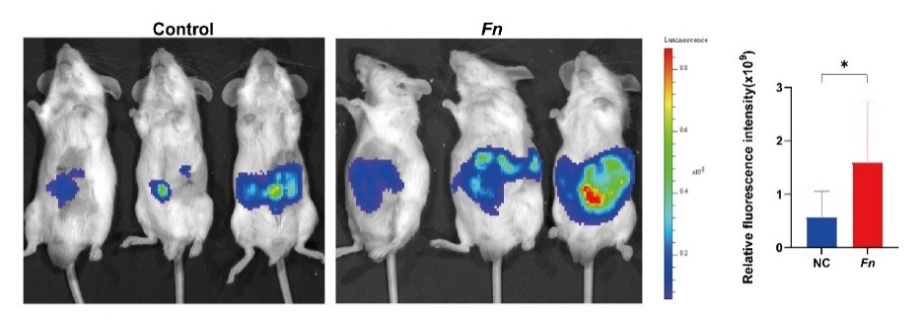


**Figure S21** IVIS images of bioluminescence signal of luciferase assay and quantitative analysis for each group. CT26-Luc cells (Control) and *Fn*-infected CT26-Luc cells were injected subcutaneously into the spleen of mice and observed at day 14(n=3). * *P* < 0.05; ** *P* < 0.01; *** *P* < 0.001.

**Figure S22**


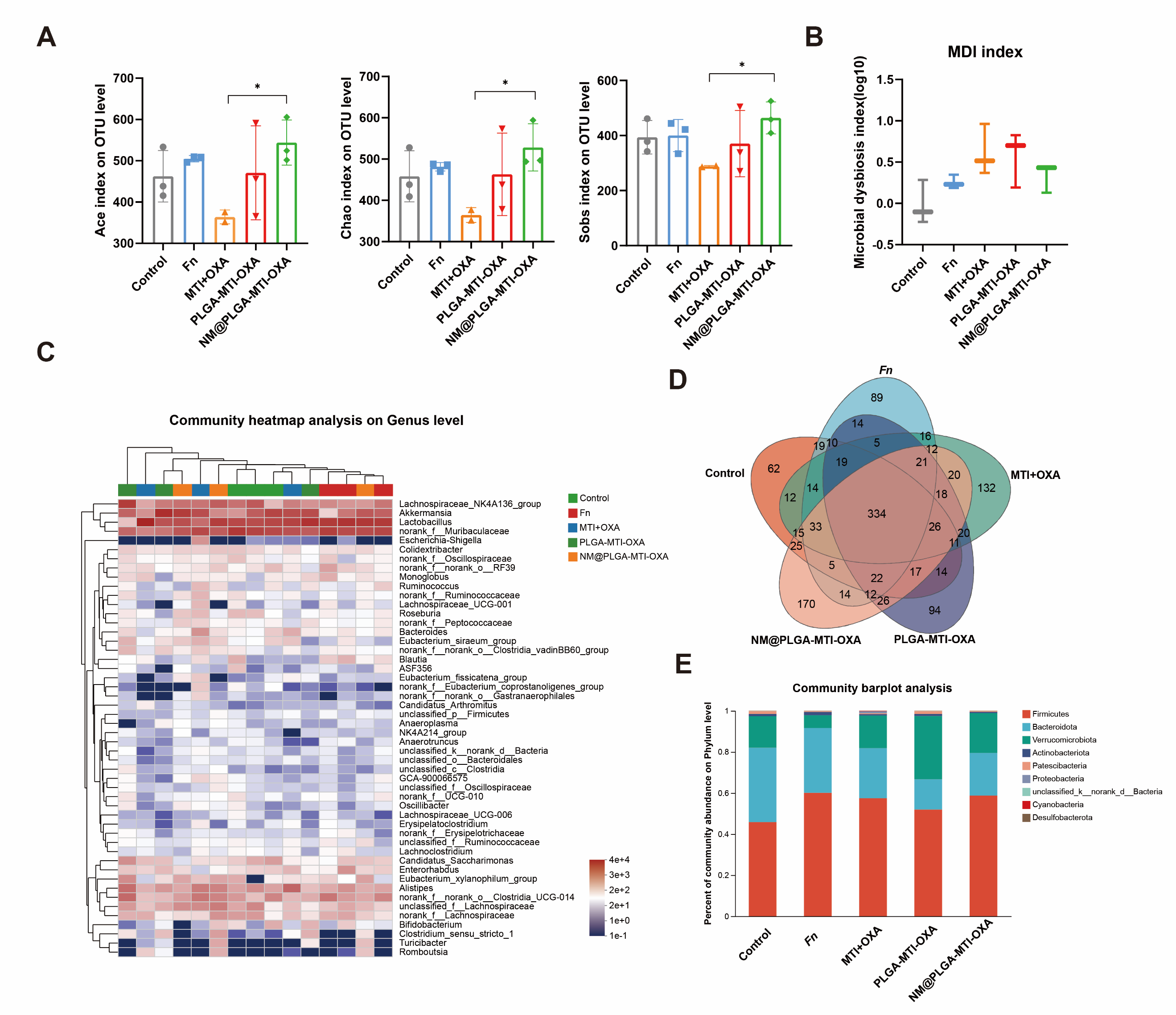


**Figure S22** Neutrophil-mimicking nanoparticles maintained intestinal flora balance in MC38/*Fn* axillary colorectal cancer model.**(A)** Analysis of alpha diversity of intestinal flora, ace index, chao index and sobs index was observed by 16S rDNA sequencing. **(B)** Microbiota dysbiosis index (MDI) of different group. **(C)** Community heatmap analysis on genus level was performed to explore the differences in intestinal flora composition after different drug treatments. **(D)** Venn analysis of species in different group. **(E)** Stacked bar plot of the phylum level relative abundance of bacteria communities in indicated samples. * *P* < 0.05; ** *P* < 0.01; *** *P* < 0.001.

**Figure S23**


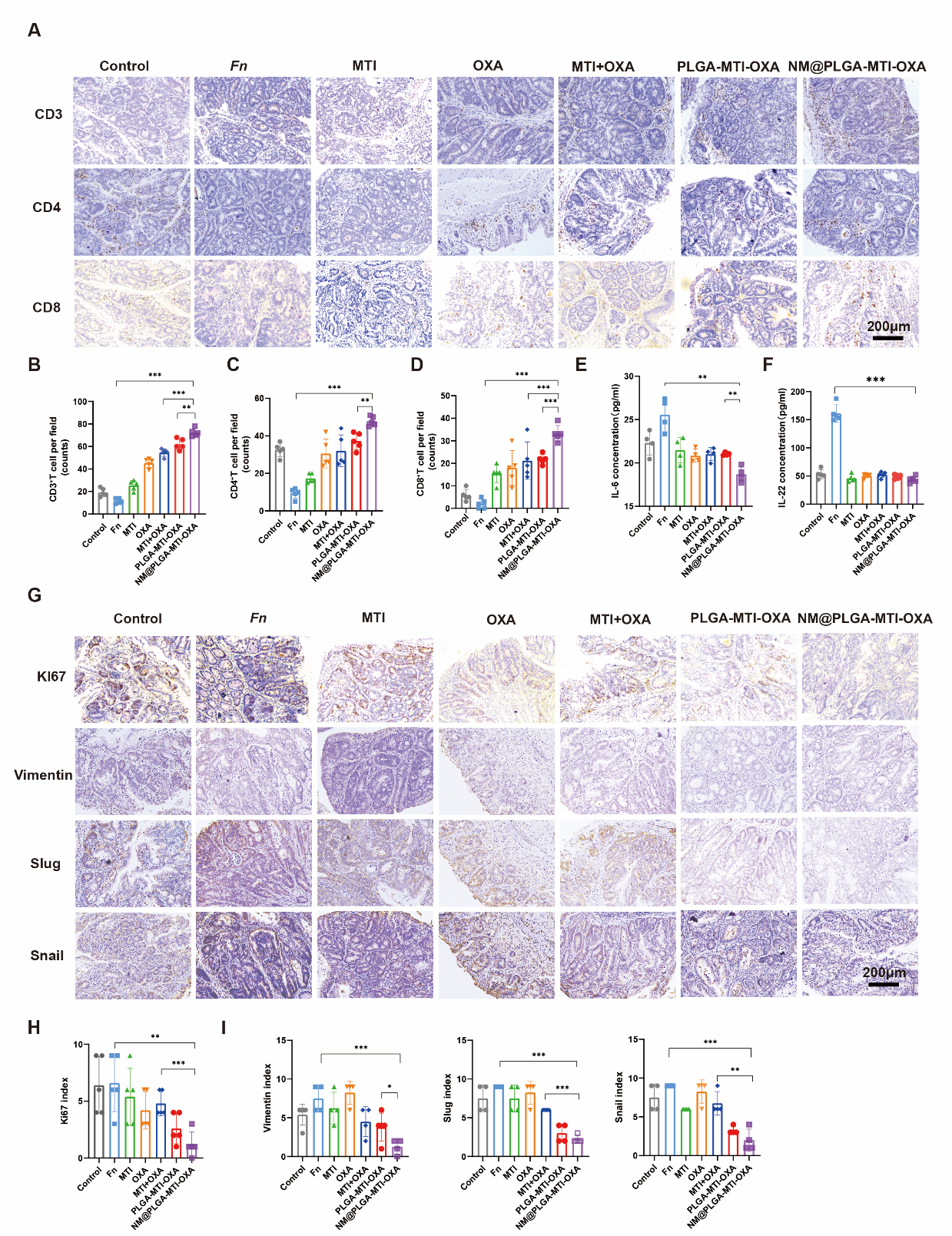


**Figure S23** Neutrophil-mimicking nanoparticles remodeled the tumor immune microenvironment and reversed the *Fn*-mediated EMT process in AD/*Fn* CRC spontaneous model. **(A)** Representative IHC staining images of CD3, CD4, CD8 in AD/*Fn* CRC spontaneous model. Scale bar = 200 μm. **(B, C, D)** Quantitative analysis in A(n=5). **(E, F)** Cytokine levels of IL-6, and IL-22 in serum were detected through ELISA among different treated groups. **(G)** Representative IHC staining images of Ki67, N-cadherin, slug, and snail in AD/*Fn* CRC spontaneous model. Scale bar = 200 μm. **(H, I)** Quantitative analysis in G. * *P* < 0.05; ** *P* < 0.01; *** *P* < 0.001.

**Figure S24**


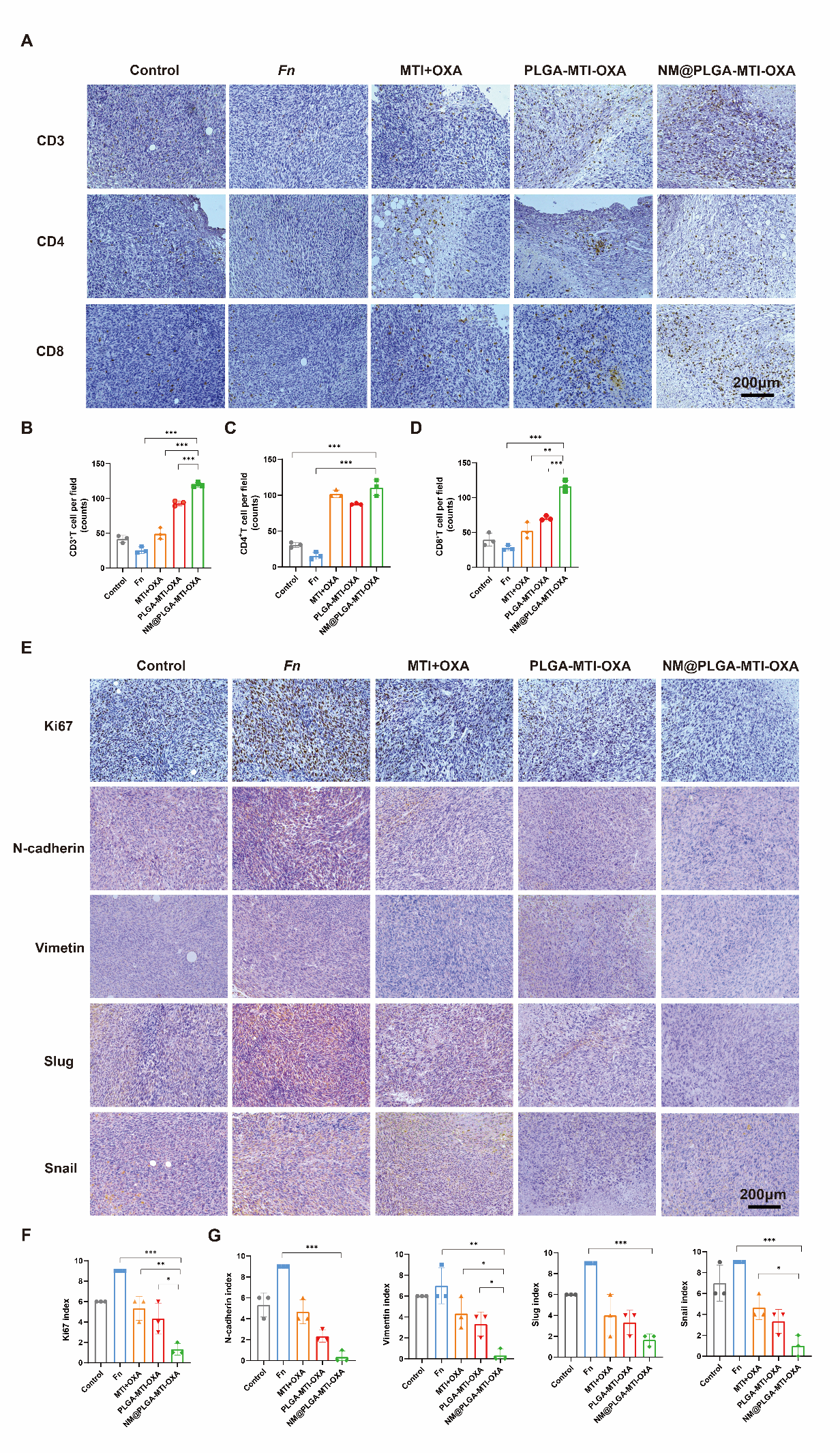


**Figure S24** Neutrophil-mimicking nanoparticles remodeled the tumor immune microenvironment and reversed the *Fn*-mediated EMT process in MC38/*Fn* axillary colorectal cancer model. **(A)** Representative IHC staining images of CD3, CD4, CD8 in MC38/*Fn* axillary colorectal cancer model. Scale bar = 200 μm. **(B, C, D)** Quantitative analysis in A. **(E)** Representative IHC staining images of Ki67, N-cadherin, Vimentin, Slug, and Snail in in MC38/*Fn* axillary colorectal cancer model. Scale bar = 200 μm. **(F, G)** Quantitative analysis in E(n=3). * *P* < 0.05; ** *P* < 0.01; *** *P* < 0.001.

**Figure S25**


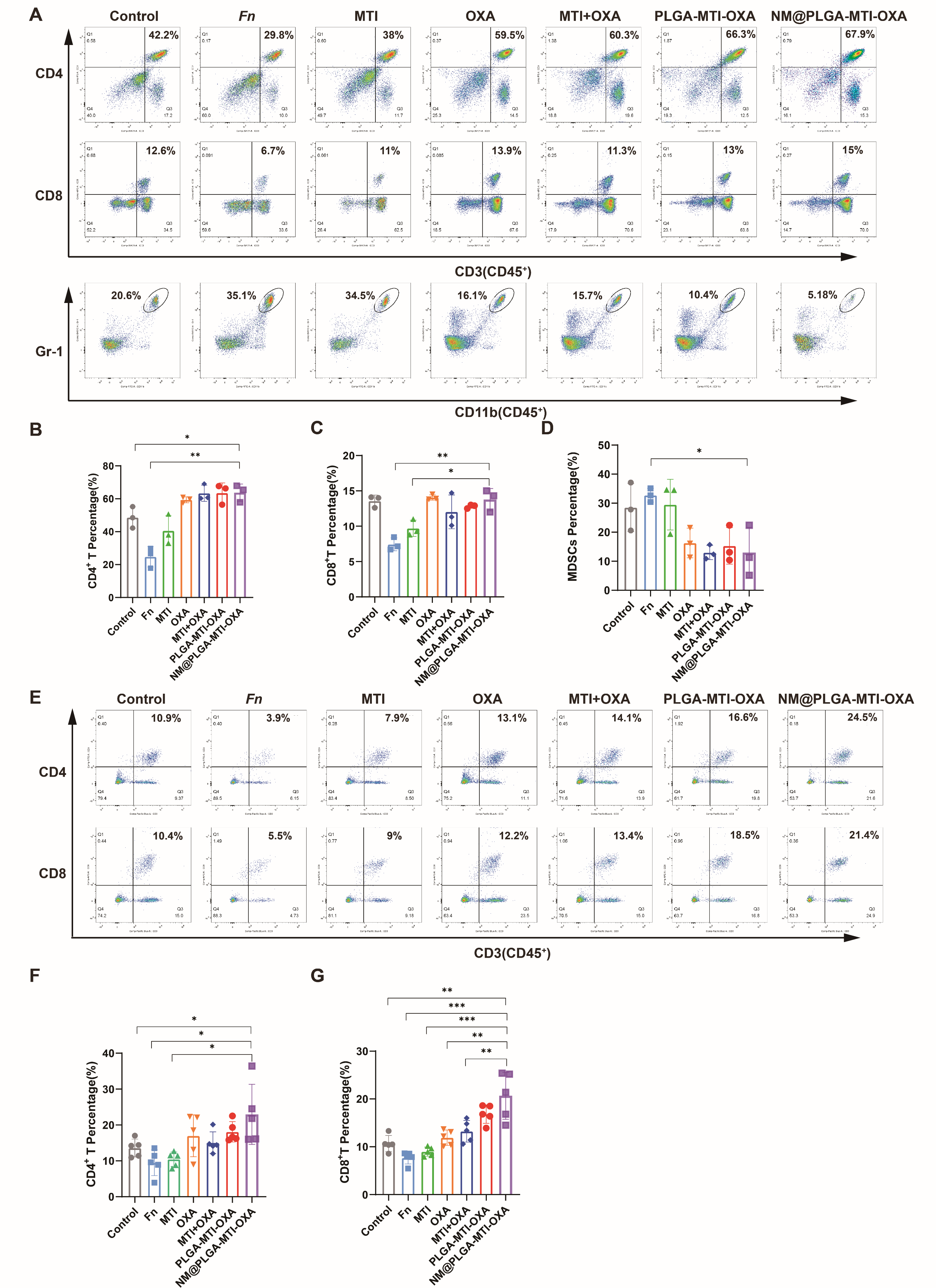


Figure S25 Flow cytometry in peripheral blood of different models.**(A)** The percentages of CD4^+^ T, CD8^+^ T cells, and MDSCs in *Fn*-infected liver metastasis model mouse peripheral blood among different treatments. **(B, C, D)** Quantitative analysis in A(n=3). **(E)** The percentages of CD4^+^ T, and CD8^+^ T cells in AD/*Fn* CRC spontaneous model mouse peripheral blood among different treatments. **(F, G)** Quantitative analysis in E (n=5). * *P* < 0.05; ** *P* < 0.01; *** *P* < 0.001.

**Table S1: Correlation between *Fn* enrichment level and clinicopathological characteristics in CRC cases**

| Variable | Total | *Fn* | | *p* value | R |
| --- | --- | --- | --- | --- | --- |
|  |  | high | low |  |  |
| Age (years) |  |  |  |  |  |
| <50 | 7 | 4 | 3 | 0.684 | -0.098 |
| ≥50 | 29 | 13 | 16 |  |  |
| Sex |  |  |  |  |  |
| female | 11 | 3 | 8 | 0.156 | 0.155 |
| male | 25 | 14 | 11 |  |  |
| Depth of invasion |  |  |  |  |  |
| T1+T2 | 3 | 3 | 0 | 0.095 | -0.319 |
| T3+T4 | 33 | 14 | 19 |  |  |
| Lymph node stage |  |  |  |  |  |
| N0 | 14 | 7 | 7 | 1.00 | -0.44 |
| N1 | 22 | 10 | 12 |  |  |
| Metastasis stage |  |  |  |  |  |
| M0 | 18 | 3 | 15 | **0.001** | **0.612** |
| M1 | 18 | 15 | 3 |  |  |
| Stage |  |  |  |  |  |
| Ⅰ+Ⅱ | 8 | 1 | 7 | **0.044** | **0.372** |
| Ⅲ+Ⅳ | 28 | 16 | 12 |  |  |
